# Antimicrobial drug use in the first decade of life influences saliva microbiota diversity and composition

**DOI:** 10.1186/s40168-020-00893-y

**Published:** 2020-08-21

**Authors:** Sajan C. Raju, Heli Viljakainen, Rejane A. O. Figueiredo, Pertti J. Neuvonen, Johan G. Eriksson, Elisabete Weiderpass, Trine B. Rounge

**Affiliations:** 1 Folkhälsan Research Center, Topeliuksenkatu 20, 00250 Helsinki, Finland; 2 Faculty of Medicine, University of Helsinki, Helsinki, Finland; 3 Department of Food and Environmental Sciences, University of Helsinki, Helsinki, Finland; 4 Department of Clinical Pharmacology, University of Helsinki and Helsinki University Hospital, Helsinki, Finland; 5 Department of General Practice and Primary Health Care, University of Helsinki and Helsinki University Hospital, Helsinki, Finland; 6 Yong Loo Lin School of Medicine, National University of Singapore, Singapore, Singapore; 7 International Agency for Research on Cancer, World Health Organization, Lyon, France; 8 Department of Research, Cancer Registry of Norway, Oslo, Norway; 9 Department of Informatics, University of Oslo, Oslo, Norway

**Keywords:** Lifelong antimicrobial use, Saliva microbiota, Azithromycin, Gender difference, Antibiotics

## Abstract

**Background:**

The human microbiota contributes to health and well-being. Antimicrobials (AM) have an immediate effect on microbial diversity and composition in the gut, but next to nothing is known about their long-term contribution to saliva microbiota. Our objectives were to investigate the long-term impact of AM use on saliva microbiota diversity and composition in preadolescents. We compared the lifetime effects by gender and AMs. We used data from 808 randomly selected children in the Finnish Health In Teens (Fin-HIT) cohort with register-based data on AM purchases from the Social Insurance Institution of Finland. Saliva microbiota was assessed with 16S rRNA (V3-V4) sequencing. The sequences were aligned to the SILVA ribosomal RNA database and classified and counted using the mothur pipeline. Associations between AM use and alpha-diversity (Shannon index) were identified with linear regression, while associations between beta-diversity (Bray-Curtis dissimilarity) and low, medium or high AM use were identified with PERMANOVA.

**Results:**

Of the children, 53.6% were girls and their mean age was 11.7 (0.4) years. On average, the children had 7.4 (ranging from 0 to 41) AM prescriptions during their lifespan. The four most commonly used AMs were amoxicillin (*n* = 2622, 43.7%), azithromycin (*n* = 1495, 24.9%), amoxicillin-clavulanate (*n* = 1123, 18.7%) and phenoxymethylpenicillin (*n* = 408, 6.8%). A linear inverse association was observed between the use of azithromycin and Shannon index (*b* − 0.015, *p* value = 0.002) in all children, the effect was driven by girls (*b* − 0.032, *p* value = 0.001), while not present in boys. Dissimilarities were marked between high, medium and low users of all AMs combined, in azithromycin users specifically, and in boys with amoxicillin use. Amoxicillin and amoxicillin-clavulanate use was associated with the largest decrease in abundance of *Rikenellaceae*. AM use in general and phenoxymethylpenicillin specifically were associated with a decrease of *Paludibacter* and pathways related to amino acid degradations differed in proportion between high and low AM users.

**Conclusions:**

A systematic approach utilising reliable registry data on lifetime use of AMs demonstrated long-term effects on saliva microbiota diversity and composition. These effects are gender- and AM-dependent. We found that frequent lifelong use of AMs shifts bacterial profiles years later, which might have unforeseen health impacts in the future. Our findings emphasise a concern for high azithromycin use, which substantially decreases bacterial diversity and affects composition as well. Further studies are needed to determine the clinical implications of our findings.

Video Abstract

## Background

Humans depend on colonised microbes to assist in digestion, produce vitamins and nutrients, resist invading pathogens and regulate metabolism and the immune system, as reviewed previously [1]. Because of the microbiome’s vast impacts, it is considered the largest organ of the human body [2, 3]. Although many studies have focused on gut microbiota, since it is the most densely populated bacterial community on Earth [4], microbiota in other niches of the body have also demonstrated importance for human health and well-being.

The saliva microbiome is associated with oral health and hygiene, but previous findings suggest connections with human health beyond the oral cavity, including inflammatory bowel disease [5], metabolic syndrome [6], atherosclerosis [7], cirrhosis with hepatic encephalopathy [8], certain cancers [9, 10] and obesity [11, 12]. There are several advantages in using saliva instead of faecal samples in studies of human health, such as the temporal stability of saliva microbiota [13] due to robustness against changes in diet [14] and other exposures in the environment [13, 14], as well as the ease for people of all ages to donate. For these reasons, saliva provides several advantages over faecal samples for the development and measurement of biomarkers. Saliva microbiota has the same richness of species as elsewhere in the gastrointestinal tract, while the bacterial composition is similar to stomach fluids and placentas [15, 16].

Exposure to antimicrobial (AM) agents has been reported to cause dysbiosis in gut microbiota, which is linked to several adverse health outcomes in human and animal models [17, 18]. The short-term consequences of AMs on the gut microbiota ecology are well-established [19–23], but less is known about saliva microbiota and long-term effects. Studies have addressed the effect of a single AM dose [24], a short-term AM therapy [25] and a 10-day treatment with AM [26] on saliva microbiota diversity and composition, mainly in adults. A single dose of the most commonly used AMs (ciprofloxacin, clindamycin, amoxicillin) caused minor and short-term changes in saliva microbial profiles and metagenomes, while dissimilarities in microbiomes were still observed 1 week and 1 month after the dose of amoxicillin and clindamycin, respectively [24]. Compared with alterations in gut microbiota, these were considered short-term and superficial [24]. Similarly, saliva diversity decreased with a 3-day treatment of azithromycin in adults [25] but was recovered within the next 8 weeks, while these effects were more profound and long-lasting in the gut. On the other hand, a 10-day treatment with amoxicillin caused a decrease in saliva microbial diversity, which was not recovered within 3 weeks in children [26].

AMs are typically the first and most widely used drugs in paediatric populations [27, 28]. In fact, the highest prevalence of AM use is observed during infancy [29, 30]. AMs are suspected to modify the development of our immune system [31] and affect susceptibility to various non-communicable diseases later in life, likely through the microbiota [32]. Data on the long-term consequences of lifetime AM use on saliva microbiota in children are very sparse. The objectives of this initial study on this topic were to explore the effect of lifetime AM use on saliva microbiota diversity and composition in 11–12-year-old Finnish children. To get a better understanding, the effects are compared between four commonly used AMs and between genders. We hypothesise that with repeated AM use and despite re-establishment, small alterations will accumulate, resulting in notable, long-term changes in the microbiota.

## Results

The 16S rRNA amplicons sequencing generated ~ 148 million reads for 973 samples. The samples had a median read count of 113,345, mean read count of 148,508 and a range between 29 and 1,316,321 sequences. After filtering and alignment, an average of 48,248 assembled reads/sample were assigned to 6536 OTUs. OTUs were classified using the SILVA bacteria taxonomy. There were no significant associations between AM use up to three months prior to sampling and alpha-diversity (*p* value: Shannon 0.397; inverse Simpson 0.476) (Supplementary Figure S1). Diversity, defined by the Shannon index and inverse Simpson index, did not vary by age, gender or child’s language and between medium and high sequencing depths (Table 1). However, saliva microbiota richness differed by sequencing depth when low depth samples were included, regardless of rarefaction (*p* < 0.05). Therefore, participants with low sequence depth (*n* = 112) and missing background information (*n* = 7) were excluded. In total, microbiota profiles of 808 children were included in further analyses.

**Table 1:** Descriptive characteristics of the study participants

Groups	# children		Shannon index		Inverse Simpson	
	***N***	%	Mean (SD)	***p***	Mean (SD)	***p***
Age, years				0.191^a^		0.125^a^
11 (≤ 11.4)	223	27.6	2.96 (0.28)		10.58 (3.09)	
12+ (≥ 11.5)	585	72.4	2.93 (0.29)		10.20 (3.30)	
Gender				0.339^a^		0.519^a^
Boy	375	46.4	2.95 (0.27)		10.38 (2.97)	
Girl	433	53.6	2.93 (0.31)		10.24 (3.47)	
Language				0.189^b^		0.430^b^
Finnish	687	85.0	2.93 (0.29)		10.24 (3.28)	
Swedish	88	10.9	2.97 (0.26)		10.56 (3.12)	
Other	33	4.1	3.01 (0.28)		10.85 (2.92)	
Sequence depth				0.461^a^		0.798^a^
Medium (10,000–100,000)	553	66.1	2.95 (0.28)		10.29 (3.07)	
High (> 100,000)	255	30.5	2.93 (0.33)		10.35 (3.62)	

^a^*t* test

^b^ANOVA test

Excluded 112 samples with low sequence depth (< 10,000)

**Fig. 1:**
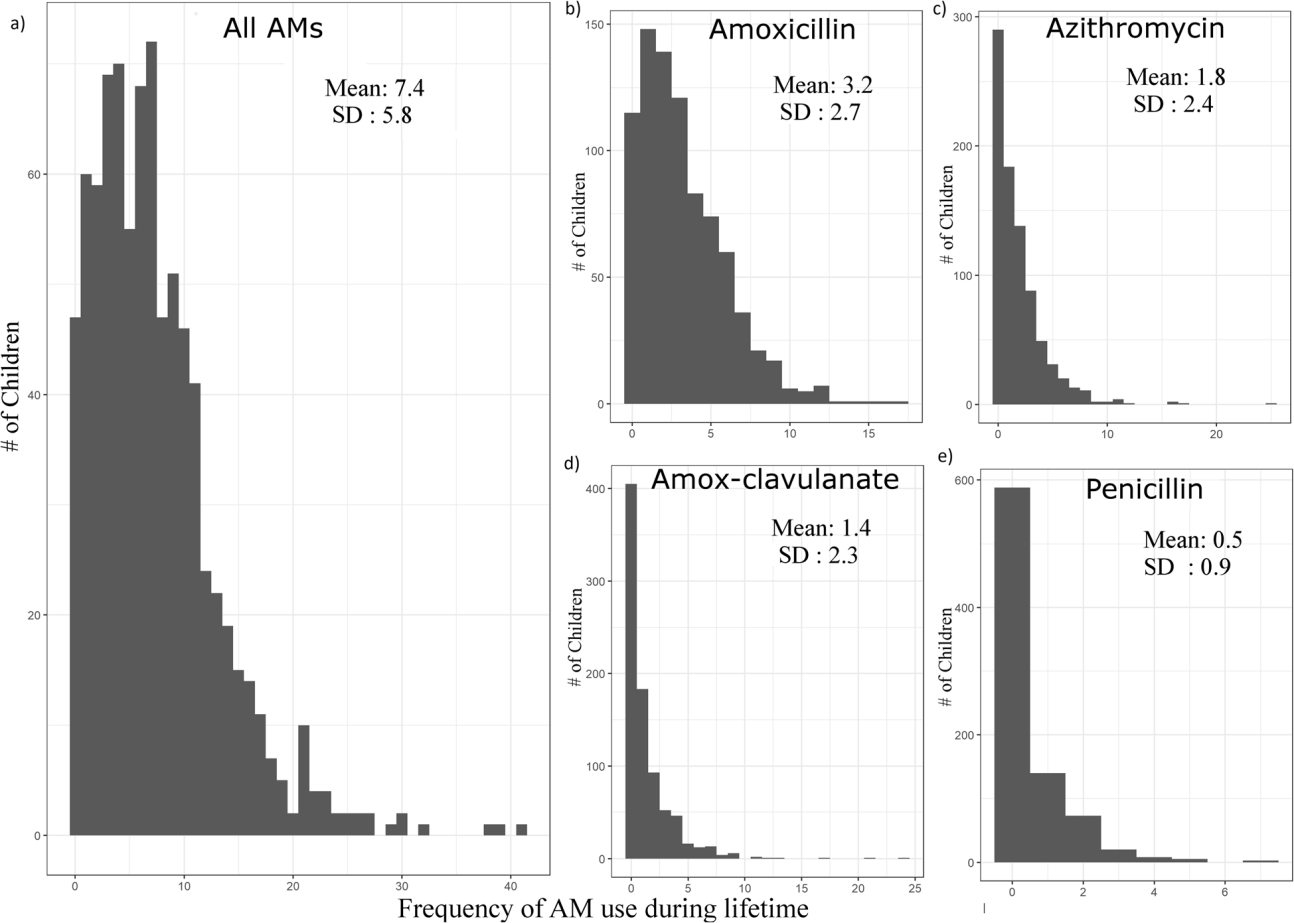
Distribution of children with purchases for **a** all antimicrobials (AMs) combined, and separately for **b** amoxicillin, **c** azithromycin, **d** amoxicillin-clavulanate and **e** phenoxymethylpenicillin during a mean follow-up time of 11.7 years. Mean and SD for the AM purchases are included in the figure

The distribution of children by the frequency of all AM use and separated by the four most commonly used AMs in the cohort—amoxicillin, azithromycin, amoxicillin-clavulanate and phenoxymethylpenicillin—are described in Fig. 1. The average number of any AM purchases was 7.4 (SD = 5.8) per child during a mean follow-up time of 11.7 (0.4) years. We did not observe gender differences in average AM use (Supplementary Figure S2). The purchases of any AMs demonstrate a decrease with age (Supplementary Figure S3). Similarly, the use of amoxicillin, azithromycin and amoxicillin-clavulanate decreased with age, while the use of phenoxymethylpenicillin peaked at the age of 7 years. Of preadolescents, 51 were AM naïve (no AM purchases) or had no registry data available. In our data, 4 participants had used any AM over 30 times.

### Antimicrobial (AM) drugs and saliva microbiota

In summary, there were 5996 AM drug regimens prescribed to 808 children during their first 12 years of life (denoted here as lifetime). The four most commonly prescribed AMs were amoxicillin (*n* = 2622, 43.7%), azithromycin (*n* = 1495, 24.9%), amoxicillin-clavulanate (*n* = 1123, 18.7%) and phenoxymethylpenicillin (*n* = 408, 6.8%).

### Alpha-diversity and lifetime AM prescriptions

With linear regression, we observed no association between prescriptions of all AMs combined and alpha-diversity assessed by the Shannon index or inverse Simpson index in all children (Table 2). We also tested the associations for the most common AM prescriptions. The models were adjusted for age, gender, language and other AM use. An inverse association of azithromycin with the Shannon index (*p* = 0.002) was observed, but no associations observed for the other three AM prescriptions. In gender-specific analyses, boys presented with an inverse association of all AMs with the Shannon index, while this was not marked in girls. Correspondingly, in girls the use of azithromycin was strongly associated with the Shannon index (*p* < 0.001): a 0.032 decrease per azithromycin course was observed, while not in boys.

**Table 2:** Associations of antimicrobial (AM) prescriptions (all AMs, and for amoxicillin, azithromycin, amoxicillin-clavulanate and phenoxymethylpenicillin AMs separately) with alpha-diversity, using the Shannon index and inverse Simpson index, in all the participants and separately in boys and girls using linear regression analysis

	Prescriptions of	All participants				Boys				Girls			
		*n*	Estimate	SE	*p*	*n*	Estimate	SE	*p*	*n*	Estimate	SE	*p*
Shannon	All AMs	5996	− 0.003	0.002	0.137	**2957**	**− 0.005**	**0.002**	**0.045**	3039	− 0.001	0.003	0.648
	Amoxicillin	2622	0.002	0.004	0.685	1280	− 0.007	0.005	0.186	1342	0.011	0.006	0.079
	Azithromycin	**1495**	**− 0.015**	**0.005**	**0.002**	783	− 0.003	0.006	0.623	**712**	**− 0.032**	**0.008**	**< 0.001**
	Amoxicillin-clavulanate	1123	0.003	0.005	0.471	531	− 0.005	0.007	0.509	592	0.011	0.007	0.093
	Phenoxymethylpenicillin	408	0.004	0.011	0.673	199	− 0.002	0.014	0.895	209	0.014	0.015	0.378
Inverse Simpson	All AMs	5996	− 0.013	0.020	0.500	2957	− 0.038	0.026	0.147	3039	0.006	0.029	0.848
	Amoxicillin	2622	0.007	0.045	0.876	1280	− 0.053	0.060	0.379	1342	0.066	0.068	0.328
	Azithromycin	1495	− 0.077	0.052	0.143	783	− 0.024	0.063	0.705	712	− 0.159	0.087	0.067
	Amoxicillin-clavulanate	1123	0.016	0.054	0.770	531	− 0.066	0.078	0.393	592	0.080	0.075	0.282
	Phenoxymethylpenicillin	408	0.077	0.118	0.513	199	0.042	0.158	0.791	209	0.135	0.174	0.439

Significant results in bold

Adjusted for age, gender (not in gender-specific analysis) and language. *SE* Std. error, *n* number of AM prescriptions

Regardless of observed gender-specific associations between AM use and the Shannon index, the AM purchase pattern for all AMs, and amoxicillin, azithromycin, amoxicillin-clavulanate and phenoxymethylpenicillin separately, by age were similar in boys and girls (Supplementary Figure S4).

**Fig. 2:**
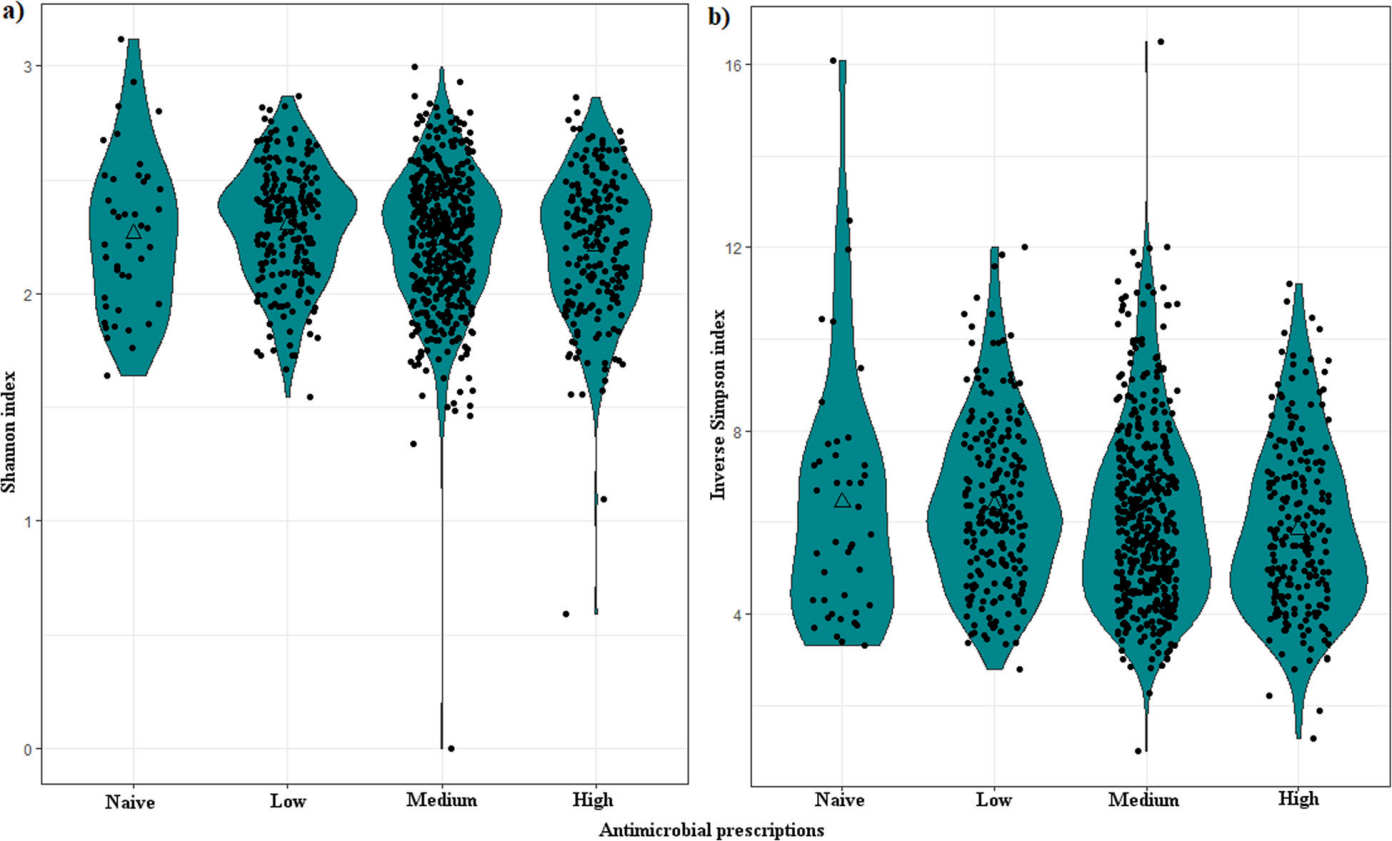
Violin plot showing the distribution of alpha-diversity as measured by **a** Shannon index and **b** inverse Simpson index for the four groups of AM use. Triangle inside the violin indicates the mean diversity

In a groupwise comparison, naïve AM users (*n* = 51) showed similar diversity with the low AM group when comparing alpha-diversity between the four AM groups (naïve, low, medium and high). Thus, naïve and low AM users were combined for further analyses (Fig. 2).

### Beta-diversity and lifetime AM use

**Fig. 3:**
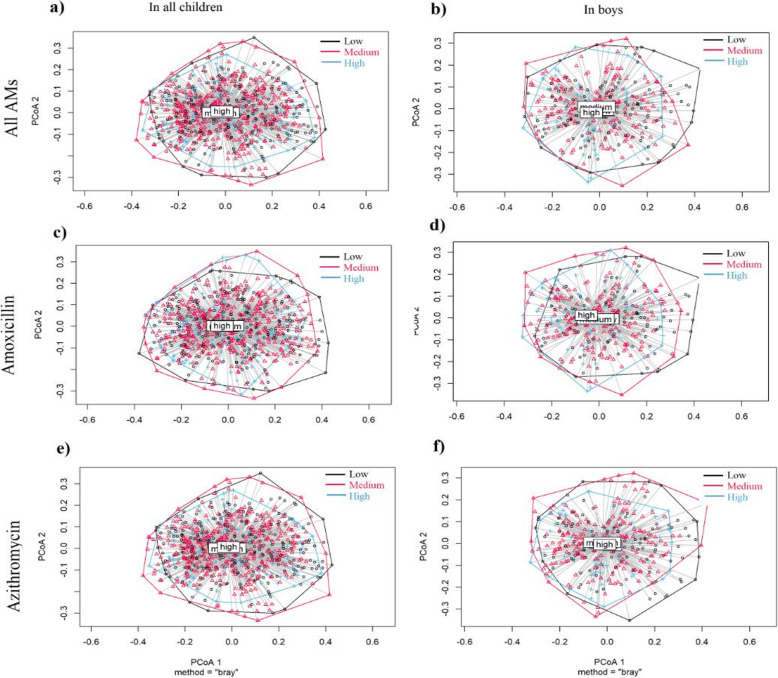
Analysis, showing the beta-dispersion, based on Bray-Curtis dissimilarity between low, medium and high groups in **a** all AMs in all children, **b** all AMs in boys, **c** amoxicillin use in all children, **d** amoxicillin use in boys, **e** azithromycin use in all children and **f** azithromycin use in boys. Permutational analysis of variance (PERMANOVA) test adjusted for age, gender (not in gender-specific analysis), language and other AMs

When comparing microbiota composition between low, medium and high users of all AMs combined, we found that beta-diversity differed between the groups (*p* = 0.033) (Table 3). Similar analyses were conducted for the most common AMs. Of these, beta-diversity differed between azithromycin groups (*p* = 0.006), especially between low and medium azithromycin users (*p* = 0.067) (Table 3). Gender-specific analysis identified a difference in microbiota composition between the groups of all AMs combined (*p* = 0.034), amoxicillin (*p* = 0.001) and azithromycin (*p* = 0.0024) in boys (Fig. 3), but no overall differences in girls (Additional file 2: Figure S5).

**Table 3:** Differences in beta-diversity, using the Bray-Curtis dissimilarity matrix, between groups of low, medium and high use of antimicrobials (AM) for all AMs combined, and separately for amoxicillin, azithromycin, amoxicillin-clavulanate and phenoxymethylpenicillin, in all children and separately for boys and girls with PERMANOVA

Prescriptions	Pairwise (***n***)	All children	Boys (***n*** = 375)	Girls (***n*** = 433)
		P	P	P
All AM		**0.033**^**a**^	**0.034**^**a**^	0.416^a^
	Low (291)–Medium (392)	0.174	0.110	0.607
	Low (291)–High (125)	0.366	0.263	0.415
	Medium (392)–High (125)	0.569	0.808	0.325
Amoxicillin		0.094^a^	**0.001**^**a**^	0.487^a^
	Low (252)–Medium (402)	0.802	*0.092*	0.944
	Low (252)–High (154)	0.352	**0.011**	0.375
	Medium (402)–High (154)	0.757	0.288	0.569
Azithromycin		**0.006**^**a**^	**0.024**^**a**^	0.273^a^
	Low (278)–Medium (399)	0.067	0.075	0.092
	Low (278)–High (131)	0.219	0.252	0.256
	Medium (399)–High (131)	0.370	0.562	0.713
Amoxicillin-clavulanate		0.256^a^	0.877^a^	0.304^a^
	Low (389)–Medium (267)	0.897	0.778	0.876
	Low (389)–High (152)	0.202	0.281	0.633
	Medium (267)–High (152)	0.455	0.421	0.437
Phenoxymethylpenicillin		0.197^a^	0.504^a^	0.574^a^
	Low (568)–Medium (135)	0.655	0.710	0.708
	Low (568)–High (105)	0.187	0.410	0.276
	Medium (135)–High (105)	0.738	0.802	0.347

Significant results in bold

Adjusted for age, gender (not in gender-specific analysis), language and depth

*p* values adjusted for all pairwise PERMANOVA analyses with Benjamini-Hochberg method

^a^PERMANOVA analysis with all (low, medium and high) AM groups

### Differentially abundant bacteria according to lifetime use of all AMs

The six most abundant bacteria phyla in the saliva microbiota for all participants were *Firmicutes* (51.9%, SD ± 11.5), *Bacteroidetes* (17.6%, SD ± 7.3), *Proteobacteria* (16.5%, SD ± 11.5), *Actinobacteria* (7.5%, SD ± 6.0), *Candidate division* TM7 (3.5%, SD ± 3.04) and *Fusobacteria* (2.8%, SD ± 2.1). We identified changes in abundance at the OTU level with respect to each increment in AM use (Table 4). When considering the use of all AMs combined, we identified two OTUs with significantly lower abundance: *Paludibacter* and unclassified bacteria *Incertae Sedis* from the family *Peptostreptococcaceae*.

**Table 4:** Differentially abundant bacteria at the OTU level per increment in AM use of (a) all antimicrobials (AMs) combined and for (b) amoxicillin, (c) amoxicillin-clavulanate and (d) phenoxymethylpenicillin, adjusted for age, gender, language and other AMs (for individual AMs)

	OTUs	baseMean	log_**2**_FC		lfcSE	padj	Nearest taxa
a	Otu000108	10.588	− 0.070	↓	0.015	0.010	*Incertae Sedis*
	Otu000130	4.859	− 0.095	↓	0.021	0.026	*Paludibacter*
b	Otu000257	0.579	− 2.378	↓	0.360	0.001	*Rikenellaceae*
c	Otu000257	0.579	− 4.852	↓	0.678	0.001	*Rikenellaceae*
d	Otu000130	4.859	− 0.216	↓	0.045	0.008	*Paludibacter*

The log_2_fold changes reported are per increment unit AM

*baseMean* mean of normalised counts of all samples, *lfcSE* standard error, *padj* adjusted *p* value

“↓” symbolises the abundances decrease per use of AM

For the most commonly purchased AMs, *Rikenellaceae* substantially decreased with increasing use of amoxicillin (log_2_FC = − 2.4) and amoxicillin-clavulanate (log_2_FC = − 4.9). The use of phenoxymethylpenicillin was associated with a decrease in abundancy of *Paludibacter* (Table 4).

### Functional prediction by PICRUSt2

**Fig. 4:**
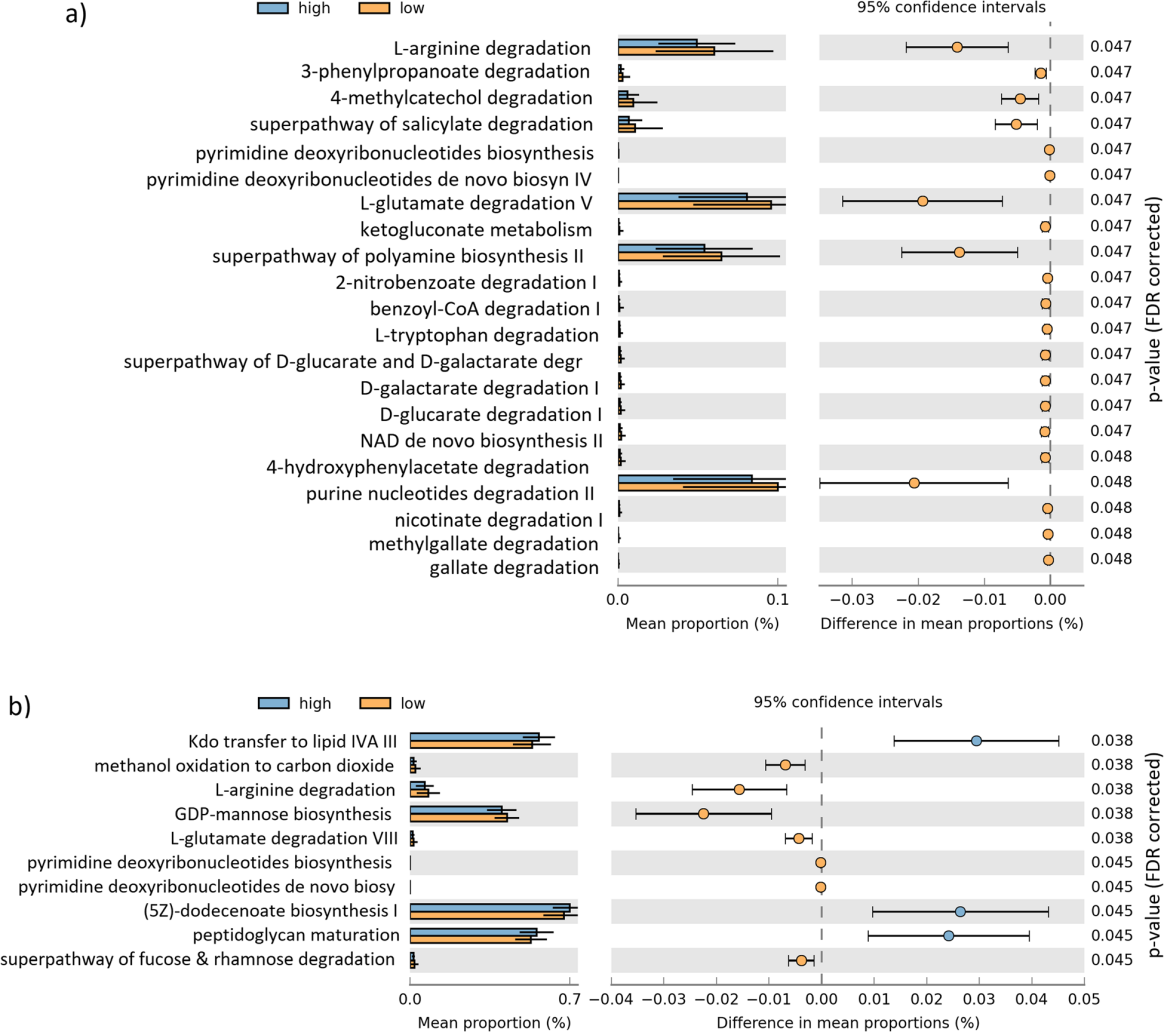
Functionally predicted MetaCyc pathways differing in proportions in high and low user groups of **a** all AMs and in **b** azithromycin. The bar plot shows mean proportions of differential MetaCyc pathways predicted using PICRUSt2. The difference in proportions between the groups is shown with 95% confidence intervals. Only *p* value < 0.05 (Welch’s *t* test, FDR adjusted), are shown

Functional predictions identified 21 differentially present metaCyc pathways between the low and high AM users when all AM use were combined (Fig. 4a). All of the pathways had higher proportions in the low AM use group. The largest significant differences were pathways for L-arginine degradation, L-glutamate degradation V, superpathway of polyamine biosynthesis II and purine nucleotides degradation II. Ten pathways differed between low and high azithromycin use (Fig. 4b). Methanol oxidation to carbon monoxide pathway, L-arginine degradation and GDP-mannose biosynthesis pathways showed higher proportions in the low azithromycin group, while Kdo transfer to lipid IVA III, (5Z)-dodecenoate biosynthesis and peptidoglycan maturation pathways showed higher proportions in the high azithromycin group.

## Discussion

Here, we describe an association of lifetime AM use with saliva microbiota richness, diversity composition and functional capacity in Finnish adolescents. AM use was obtained reliably and objectively from the medical purchase registry held by the Social Insurance Institution of Finland (www.kela.fi), which contains information on all medical expenditures and purchases requiring prescriptions. The data covers the whole lifespan of the adolescents for each of the 808 children individually. Our key findings are (i) lifelong exposure to AMs is associated with alterations of the saliva microbiota, (ii) the long-term effect of azithromycin use was the most noticeable and (iii) re-establishment of saliva microbiota occurs rapidly after AM exposure. More diverse microbiota communities are believed to be more stable and resistant towards invasion and other disturbances [33] and to sustain health. Evidently, high lifespan exposure to AMs perturbs saliva microbiota. Microbial diversity significantly differed in boys with the use of all AMs combined in their 12-year lifespan. Only azithromycin use impairs/reduces microbial richness, and this effect was driven by girls, while not seen in boys. Lifetime use of all AMs combined and azithromycin contributed with dissimilarities in microbiota composition reflected by beta-diversity, in all children. Corresponding was seen in boys only, despite a similar pattern of use in both genders, illuminating that the AMs affect microbiota composition in boys, but not in girls. A similar trend was observed for amoxicillin groups in all children, but a significant difference in microbial composition was only seen in boys.

To our knowledge, we are the first to evaluate the consequences of lifetime AM exposure on saliva microbiota in a large cohort of adolescents. Previous studies have addressed short-term or acute/immediate effects of AM use on saliva microbiota [24–26]. These studies showed the robustness and speedy recovery of saliva microbiota after perturbation caused by AMs, compared with gut microbiota. Similarly to [24], we observed saliva microbiota to reach the same bacterial richness three months after AM use as in naïve users. To us, this indicates re-establishment of the microbiota. Re-establishment depends on the growth potential of AM unaffected taxa, the introduction of taxa requiring re-seeding and their interconnections [34]. With frequent AM use, each re-establishment event after AM-induced dysbiosis might cause minute but accumulating compositional changes. These changes might in turn have long-term health consequences [35, 36]. The same processes likely also occur for other diverse microbial communities, such as in the gut, although further analyses are needed to confirm this.

Our study was conducted in apparently healthy children. In this cohort, nearly half of the AM use occurred between ages 0 and 3 years, which is line with previous reports across Europe stating high use of AMs in paediatric populations [27–30]. Children, in whom the microbiota colonisation is still ongoing, are likely more vulnerable to AM perturbation than adults with an established microbiota [37], but we did not address the timing of the first exposure to AM, nor its effects on saliva microbiota. The effect of repeated or recurrent AM use on microbiota was not evaluated either, but we can assume it to have a more vast effect if the microbiota has not yet recovered from the previous perturbation [37].

In apparently healthy children, only the lifetime use of azithromycin was associated with lower bacterial richness in saliva and enrichment of potentially pathogenic taxa, which fulfils the criteria for microbiota dysbiosis [38]. However, this did not apply to all AMs combined or separately to any other common AM in this age group. The effects of azithromycin on microbiota could be explained by its pharmacokinetic properties. Azithromycin has a very large apparent volume of distribution of 23 l/kg, which indicates extensive penetration to and accumulation in tissues. As a lipophilic drug, it penetrates freely through cell membranes and is also active against intracellular microbes. Due to its long half-life up to 5 days, its bacteriostatic effect is well sustained by once-daily dosing and continues for several days after cessation of its use [39]. Furthermore, the bioavailability after oral dosing is about 40% (i.e., a significant fraction of its oral dose may remain in the gastrointestinal tract). This could explain some of its effects on the gut microbiota. A previous Finnish study has described macrolides to induce long-term distortions for up to 24 months in the composition and function of gut microbiota [40]. Furthermore, groups with low and medium use of azithromycin showed dissimilar bacterial composition, suggesting that the composition of saliva microbiota is also affected, not only the richness. It has been shown that macrolide use enhances an increase in antibiotic resistance in the gut microbiota, which can be resolved 6–12 months after cessation of the use [40].

Amoxicillin and phenoxymethylpenicillin are the two other commonly used AMs. Both of them are bacteriocidic and have a short half-life, but as hydrophilic drugs, they are unable to passively diffuse through plasma membranes of eukaryotic cells and are inactive against intracellular microbes. Their lifetime use did not affect the richness of saliva microbiota, while groups with low and high amoxicillin use had dissimilar microbiota compositions. Characteristic for amoxicillin use was a tendency for a lower abundance of *Paludibacter,* which is reported to possess antibiotic-resistant genes [41].

One could assume that the effects of amoxicillin-clavulanate are similar to those of amoxicillin, but in fact, the lifetime use of amoxicillin-clavulanate was not related to saliva microbiota richness or diversity at adolescence. Clavulanate (clavulanic acid, an inhibitor of the beta-lactamase enzyme) prevents bacterial inactivation of amoxicillin in this combination, but the antimicrobial effect is mediated by amoxicillin. Bacteria belonging to the *Rikenellaceae* family were enriched in children with a high lifetime use of amoxicillin-clavulanate. The only narrow-spectrum agent evaluated here was phenoxymethylpenicillin, which was not associated with richness or diversity of saliva microbiota in adolescents.

Associations of lifetime AM use with saliva microbiota varied by gender: especially the richness was affected in girls, while boys were more prone to dissimilar microbiota compositions. Previously, AMs have been associated with higher growth and weight gain in boys than in girls [42]. In addition, we have previously shown that gender differences over-rule associations between BMI and saliva microbiota [11]. In that study, alpha-diversity was dissimilar between normal-weight and obese boys and between normal- and overweight girls. The beta-diversity differed between normal-weight and obese girls, but not in boys. Our results point out that high lifelong exposure to AMs perturbs microbiota in saliva and possibly in other niches of the body and most likely contributes to the development of obesity.

We found that several pathways differed in all AM users, notably, arginine and glutamate degradation, polyamine biosynthesis pathway, methyl catechol, salicylate and hydroxy phenylacetate degradation. Children with high lifetime AM use had lower proportions of the pathways reducing L-arginine. It has been suggested that the amino acid L-arginine can inhibit the bacterial coaggregation and modify the bacterial metabolism and drug resistance in the oral cavity [43]. Glutamate plays a vital role in metabolic processes in bacterial cells and has been shown to be an important virulent factor [44]. When we considered high and low users of azithromycin, we found mannose biosynthesis, peptidoglycan maturation, dodecenoate biosynthesis and arginine degradation pathways to differ in proportion. To note, cell wall peptidoglycan is essential for the maintenance of cellular viability [45]. Moreover, it has been shown that the peptidoglycan maturation enzymes may affect the flagellar functionality [46].

Our study has several strengths, but also limitations. Our study utilises validated national registers that were available from a large cohort of children and allowed us to explore lifetime exposure to AMs objectively. However, we could not confirm that the AMs were actually taken. Diagnoses were unknown, but similar patterns of use were observed in boys and girls. Dental status was not assessed. Dentition and active or recurrent caries might confound our results [47]. Here, we were interested in lifetime use of AMs, and the timing of AM use was not incorporated in the models, but only used as descriptive data. Both early and recent use (within the last 12 months) cause alterations in the gut microbiota [48]; thus, our decision to exclude recent users was justified and an even more conservative approach could be used. The majority of children (85%) in our study were exposed to AMs during the first three years of life. To study early vs later life AM exposure would require new sampling in our study. Timing of use, and especially repeated or recurrent use, was not fully addressed here, and these warrant further studies.

## Conclusions

We have utilised reliable national registry data to demonstrate the long-term effect of AM use on saliva microbiota diversity and composition. These effects are gender- and AM-dependent. We observed that saliva microbiota diversity was inversely associated with the lifetime use of azithromycin in all children, more strongly in girls than boys. Microbiota composition differed significantly after lifetime use of all AMs combined and azithromycin in all children and a similar trend was seen in amoxicillin. Regardless of the similar pattern of AM use in both genders, differences in microbiota composition were profoundly seen in boys only. We proved that a frequent lifelong use of AM shifts bacterial profiles in saliva, which might have unforeseen health impacts in the future. Further studies are needed to confirm the clinical implications of our findings.

## Methods

### Study design and sampling

We randomly selected 973 children from the prospective Finnish Health in Teens (Fin-HIT) cohort [49] using a function written in the PHP programming language on the SQL database study database. The function selected participants and samples randomly among the list of Fin-HIT participants who took part in the school assessment, with sample delivered to the biobank and had completed the consent form at the time of recruitment. We did not include the participants from the pilot recruitment that was done at home. This cohort consists of approximately 11,000 9–14-year-old children and 6500 of their mothers or other legal guardians that were recruited in densely populated areas across Finland. During the baseline data collection, subjects filled in an online questionnaire on lifestyle factors, provided a saliva sample and had their anthropometric measures measured in a standardised way [50]. Topics covered by the child and parental questionnaires are listed in the cohort profile [49]. Unstimulated saliva samples were collected using Oragene® DNA self-collection kits (OG-500, DNA Genotek Inc., Canada) [51]. Participants mixed the saliva specimens with a stabilising reagent within the collection tube according to the manufacturer’s instructions. The samples were stored at room temperature until analyses. The samples without information on age or AM prescriptions, or withdrew consent (*n* = 7) were omitted from the analysis. Information on the child’s language or mother tongue (Finnish, Swedish, or other) was obtained from the consent forms or questionnaires and confirmed by linkage with the National Population Information System at the Population Register Centre. The language is considered to reflect the socio-economic status and ethnic background of the participant [52].

In Finland, AM agents for systemic use are available by prescription and sold solely in registered pharmacies. All drug purchases based on prescriptions are registered in the Drug Prescription Register held by the Social Insurance Institution of Finland (KELA, www.kela.fi). Information on drug purchases during the entire lifespan was linked to the Fin-HIT study database. We extracted information on all systemic antibiotics based on anatomic-therapeutic chemical (ATC) codes (WHO Collaborating Centre for Drug Statistics Methodology; https://www.whocc.no/atc_ddd_index). Information on antibiotics administered in hospitals was not collected. In this study, we use data on AM purchases from the date of birth to the date of saliva sampling as a proxy of AM use. Among all the AMs, amoxicillin (ATC-code, J01CA04), azithromycin (J01FA10), amoxicillin-clavulanate (enzyme inhibitor) (J01CR02) and phenoxymethylpenicillin (J01CE02) were the four most commonly prescribed AMs in this study population.

### Ethical aspects

The Coordinating Ethics Committee of the Hospital District of Helsinki and Uusimaa has approved the Fin-HIT study protocol (169/13/03/00/10). Subjects and one of their parents have given written informed consent, which allows us to integrate the national health register data as a part of the research material. Participants may withdraw their consent whenever they wish to.

### Amplification and sequencing

A DNA extraction protocol that contained an intensive lysis step using a cocktail of lysozyme and mechanical disruption of the bacterial cells using bead-beating was conducted at the Technology Centre, Sequencing Unit, in the Institute for Molecular Medicine Finland (FIMM), as described previously [53]. Sample amplification and sequencing libraries were prepared according to an in-house 16S PCR amplification protocol [53]. 16S primers S-D-Bact-0341-b-S-17 (5′CCTACGGGNGGCWGCAG′3) and S-D-Bact-0785-a-A-21 (5′GACTACHVGGGTATCTAATCC′3) were used to amplify the V3-V4 regions [54]. Amplification was performed using the Truseq (TS)-tailed1-step amplification protocol [53]. The sequencing of PCR amplicons was performed using the 2 × 270 bp sequencing on the Illumina HiSeq1500 instrument (Illumina, Inc., San Diego, CA, USA) at FIMM. Samples, together with nine blank samples (negative control) [53] and two water samples, were sequenced at 270-bp paired-end reads, providing sufficient overlap of high-quality sequences between the forward and reverse reads, thus reducing the error rates and providing reproducible results.

### Bioinformatics analysis

Sequencing quality filtering was carried out and sequences were processed using the MiSeq SOP in the mothur pipeline (Version v.1.35.1) [55], as previously described [53]. We used the SILVA 16S rRNA database (Version V119) and taxonomy for the alignment and classification of the sequences [56]. To ensure high-quality data for the analysis, sequence reads containing ambiguous bases, homopolymers > 8 bp, more than one mismatch in the primer sequence, less than 10 base pair assembly overlap or sequences under the default per base quality score in mothur were removed. Assembled reads > 460 bp in length and singletons were excluded from the analysis. This reduced the number of assembled reads, ensuring high-quality data [11].

The high-quality assembled reads were aligned to the SILVA 16S rRNA database, clustered into operational taxonomic units (OTUs) at a cut-off value > 98% and assigned taxonomy to OTUs using the SILVA bacteria taxonomy. OTUs were normalised by subsampling with a threshold of 2000 OTU counts excluding the minimum number of samples (*n* = 83). Low sequencing depth samples (< 10,000) were omitted from the analysis (*n* = 29). Alpha-diversity (Shannon index and inverse Simpson index) was calculated per sample. Beta-diversity (i.e., the variation in community composition between microbiota samples) was calculated and compared between the AM user groups (low, medium, high) using the Bray-Curtis dissimilarity matrix.

### Statistical analysis

All statistical analyses were conducted in R (Version 3.4.2) using the stats4 (Version 3.4.2), vegan (version 2.5-4) and phyloseq (version 1.25.2) packages. We also identified potential confounding with age and gender using *t* test and between child languages (Finnish, Swedish and other); sequence depth groups (medium and high) with ANOVA. Differences in the microbial alpha-diversity between the four groups (AM naïve (*n* = 48), used AMs 1-month (*n* = 15), 2-months (*n* = 13) and 3-months (*n* = 13) prior to saliva sampling) were evaluated using ANCOVA (adjusted for depth). Based on this result, we excluded recent (3-months) AM users from the main analysis. We also excluded participants with low sequence depth (*n* = 112) and missing background information (*n* = 7). For all further analysis, the study group consists of 808 children. Our cohort does not consist of adults, to emphasise that we use boy and girl than male and female in gender.

To study the burden of AM use in the first decade of life, we used an increment in AM purchases as a continuous variable. Association of this increment in AM purchases with alpha-diversity was evaluated using linear regression analysis. We carried out models evaluating all AMs combined and the four most common AMs separately. All models were adjusted for age, gender and language. This was performed for the whole cohort and separately for boys and girls.

Comparisons of composition were done groupwise with the Bray-Curtis dissimilarity index. Prescriptions of all AMs were divided into quartiles: the first quartile as low (≤ 4 use), second and third quartiles combined as medium (5–13 use) and fourth quartile as high (≥ 14 use) prescription groups. Similarly, the corresponding cut-offs for amoxicillin use were (< 1; 2–5; > 6), for azithromycin (0; 1–3; > 4), for phenoxymethylpenicillin (< 0; 1; > 2) and for amoxicillin-clavulanate (0; 1–2; > 3). Beta-diversity was analysed with the permutational multivariate analysis of variance test (PERMANOVA) using the adonis function in the vegan package with 999 permutations. The analysis adjusted for age, gender, language and other AMs. PERMANOVA was run for all AMs combined and for the four most common AMs separately, as defined previously. Pairwise PERMANOVA was used for multiple comparisons between prescription groups (low-medium, low-high and medium-high) and *p* values were adjusted with the Benjamini-Hochberg method.

The association of bacteria abundance at the operational taxonomic level (OTUs) was tested using General Linear models (GLM) with a Negative Binomial distribution, implemented in the DESeq2 package [57] with AM use as a continuous variable and the resulted log_2_fold change is per increment unit AM. Analyses were evaluated by (a) all AMs, (b) amoxicillin, (c) azithromycin, (d) amoxicillin-clavulanate and (e) phenoxymethylpenicillin. Rare OTUs (summarised to < 20 counts in all samples) were filtered out in DESeq2 analysis. Analyses were adjusted for age, gender, language and other AMs. We report log_2_fold change per unit of change of AM purchase, i.e., per increment unit AM. The metagenome functional profiling was predicted using Phylogenetic Investigation of Communities by Reconstruction of Unobserved States—PICRUSt2 (v2.0.0-b.2) [58], with sequences and count file after preclustering from the mothur pipeline as input. Pathways were predicted using the MetaCyc database. Differentially present pathways between low and high AM groups were analysed with welch test using STAMP (Version 2.1.3) [59]. Differentially present pathways with FDR adjusted *p* value < 0.05 was presented.

## Supplementary information

**Additional file 1: Figure S1:** Violin plot showing the distribution of alpha-diversity as measured by a) Shannon index and b) inverse Simpson index for recent antimicrobial (AM) users: 1 month, 2 months and 3 months prior to saliva sampling and in children who have never used AMs. Triangles inside the plots shows the mean diversity in the group, and these did not differ between months (ANOVA p = 0.397 and 0.476 for Shannon and Inverse Simpson, respectively). **Figure S2**: Bar plot showing the use of a) all Antimicrobials (AM) combined and separately for b) Amoxicillin, c) Azithromycin, d) Amoxicillin-clavulanate and e) Phenoxymethylpenicillin in boys and girls. **Figure S3.** Histogram showing the use of a) all antimicrobials (AMs) combined, and separately for b) Amoxicillin, c) Azithromycin, d) Amoxicillin-clavulanate, and e) Phenoxymethylpenicillin by age in all 837 children. **Figure S4.** Histogram showing the use of a) all antimicrobials (AMs) combined and separately for b) Amoxicillin, c) Azithromycin, d) Amoxicillin-clavulanate, and e) Phenoxymethylpenicillin with age separated by gender.

**Additional file 2:** **Figure S5**. Analysis showing the distance to centroid and beta-dispersion, based on Bray-Curtis dissimilarity between all AMs in a) all children and four AMs separately; and in b) boys and c) girls separately. Permutational analysis of variance (PERMANOVA) test adjusted for age, gender (not in gender-specific analysis) and language.

## Data Availability

Data will be available at the EGA database (accession number EGAS00001003039).
