# Supplementary material for: Antimicrobial drug use in the first decade of life influences saliva microbiota diversity and composition
Source: Microbiome. 2020 Aug 21;8:121. doi: 10.1186/s40168-020-00893-y (PMC7441731; doi:10.1186/s40168-020-00893-y)
Supplement: Supplementary file 2 — Additional file 1: Figure S1: Violin plot showing the distribution of alpha-diversity as measured by a) Shannon index and b) inverse Simpson index for recent antimicrobial (AM) users: 1 month, 2 months and 3 months prior to saliva sampling and in children who have never used AMs. Triangles inside the plots shows the mean diversity in the group, and these did not differ between months (ANOVA p = 0.397 and 0.476 for Shannon and Inverse Simpson, respectively). Figure S2: Bar plot showing the use of a) all Antimicrobials (AM) combined and separately for b) Amoxicillin, c) Azithromycin, d) Amoxicillin-clavulanate and e) Phenoxymethylpenicillin in boys and girls. Figure S3. Histogram showing the use of a) all antimicrobials (AMs) combined, and separately for b) Amoxicillin, c) Azithromycin, d) Amoxicillin-clavulanate, and e) Phenoxymethylpenicillin by age in all 837 children. Figure S4. Histogram showing the use of a) all antimicrobials (AMs) combined and separately for b) Amoxicillin, c) Azithromycin, d) Amoxicillin-clavulanate, and e) Phenoxymethylpenicillin with age separated by gender. [file 40168_2020_893_MOESM1_ESM.docx]

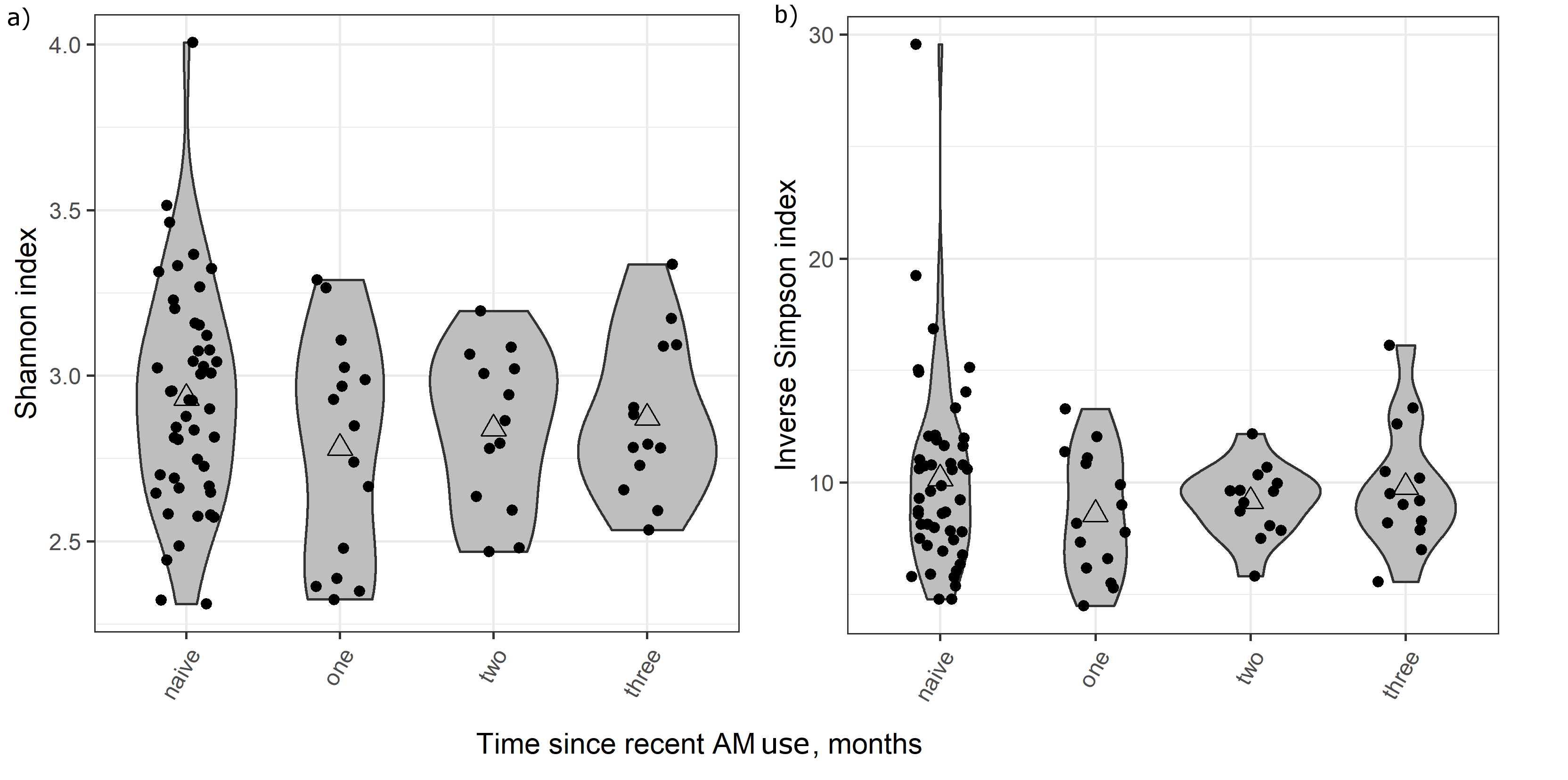


**Figure S1:** Violin plot showing the distribution of alpha diversity as measured by a) Shannon index and b) inverse Simpson index for recent antimicrobial (AM) users: 1 month, 2 months and 3 months prior to saliva sampling and in children never used AM. Triangles inside the plots shows the group mean value and these did not differ between months (ANOVA p=0.397 and 0.476 for Shannon and Inverse Simpson, respectively).


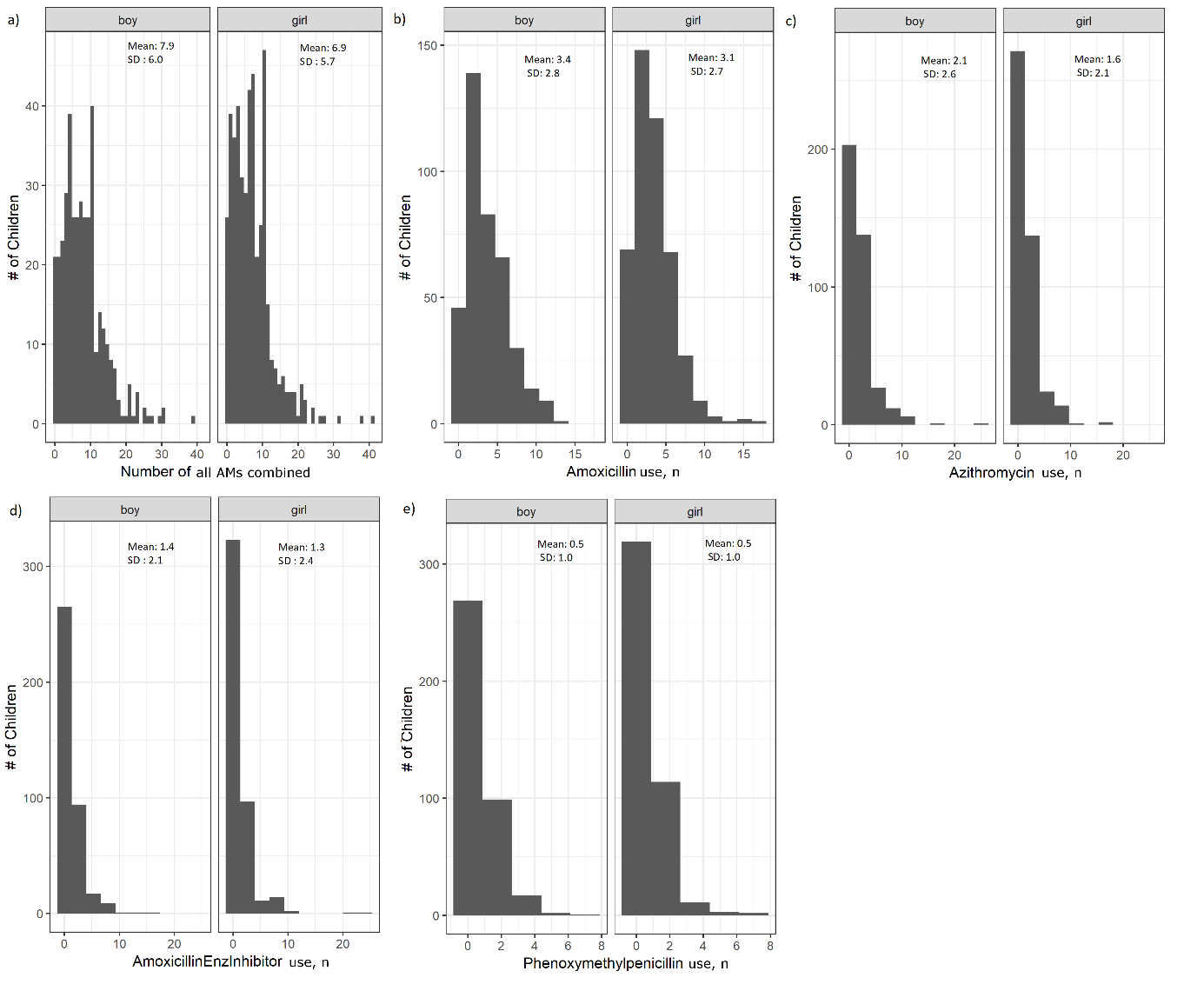


**Figure S2**: Bar plot showing the use of a) all Antimicrobials (AM) combined and separately for b) Amoxicillin, c) Azithromycin, d) Amoxicillin-clavulanate and e) Phenoxymethylpenicillin in boys and girls.


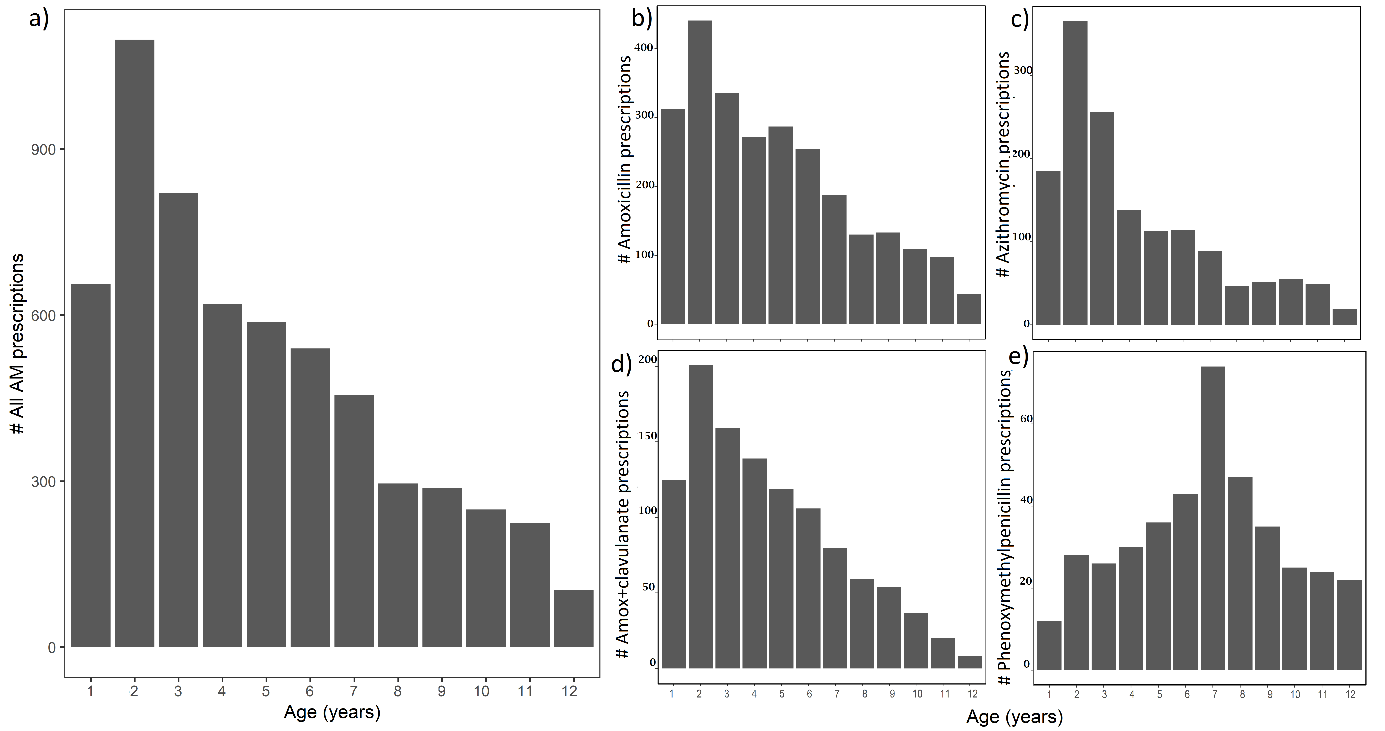


**Figure S3.** Histogram shows the use of a) all antimicrobials (AMs) combined, and separately for b) Amoxicillin, c) Azithromycin, d) Amoxicillin-clavulanate, and e) Phenoxymethylpenicillin by age in all 808 children.


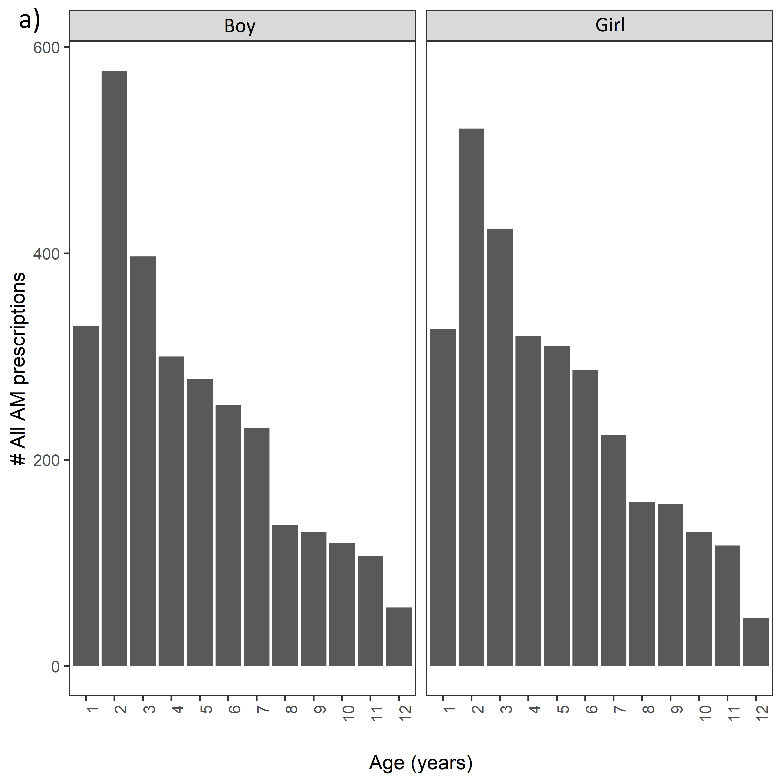


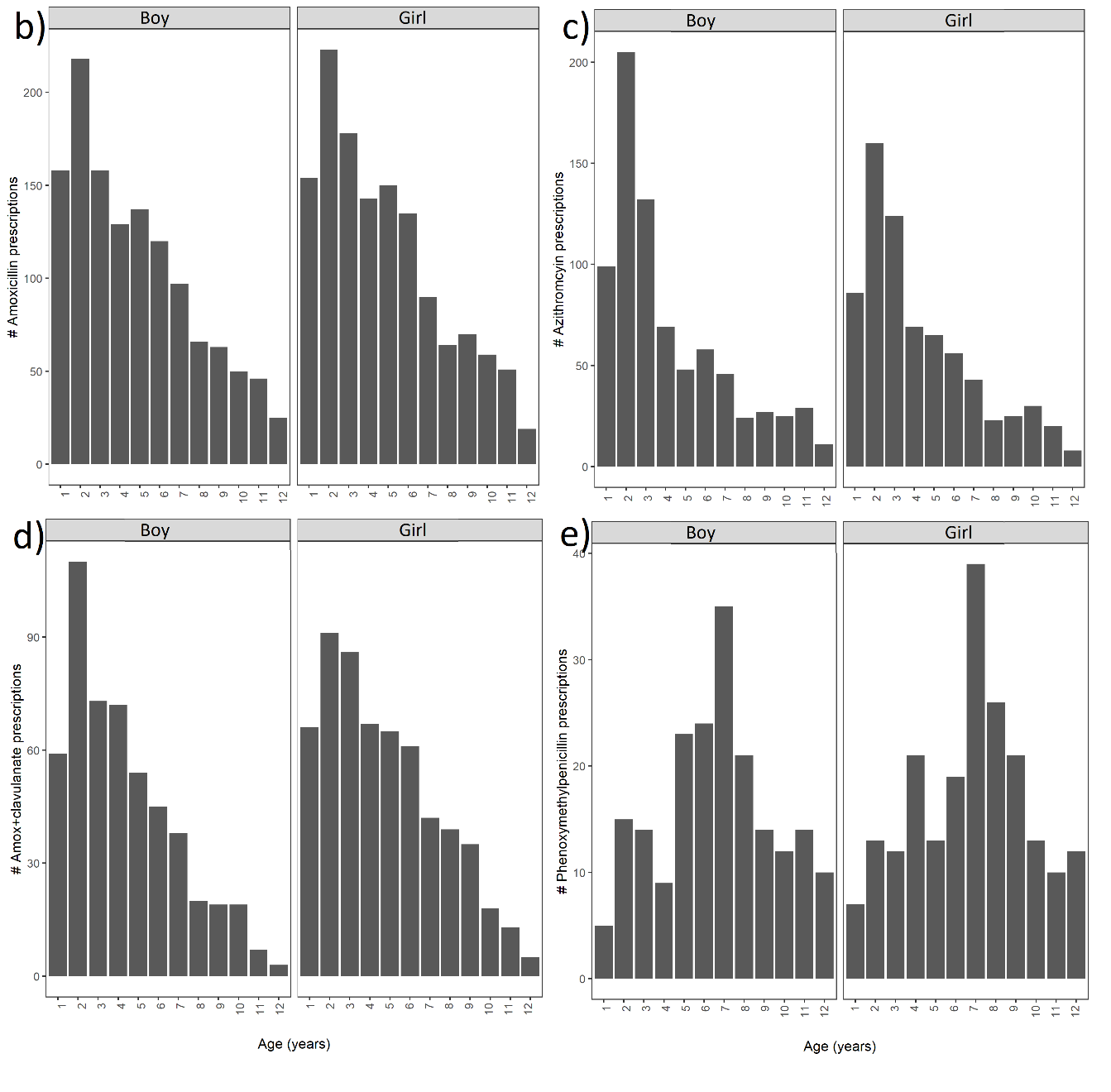


**Figure S4.** Histogram shows the use of a) all antimicrobials (AMs) combined and separately for b) Amoxicillin, c) Azithromycin, d) Amoxicillin-clavulanate, and e) Phenoxymethylpenicillin with age separated by gender.
