# Supplementary material for: Antimicrobial drug use in the first decade of life influences saliva microbiota diversity and composition
Source: Microbiome. 2020 Aug 21;8:121. doi: 10.1186/s40168-020-00893-y (PMC7441731; doi:10.1186/s40168-020-00893-y)
Supplement: Supplementary file 3 — Additional file 2: Figure S5. Analysis showing the distance to centroid and beta-dispersion, based on Bray-Curtis dissimilarity between all AMs in a) all children and four AMs separately; and in b) boys and c) girls separately. Permutational analysis of variance (PERMANOVA) test adjusted for age, gender (not in gender-specific analysis) and language. [file 40168_2020_893_MOESM2_ESM.docx]

**Additional file 2: Figure S5:** Analysis, showing the distance to centroid and beta-dispersion, based on Bray–Curtis dissimilarity between All AMs in a) all children and four AMs separately; and in b) boys and c) girls separately. Permutational analysis of variance (PERMANOVA) test adjusted for gender (not in gender specific analysis), age and language.

a)

| **AMs for all children** | **Distance centroid** | **Beta dispersion (**Low, Medium, High**)** |
| --- | --- | --- |
| Amoxicillin-clavulanate | 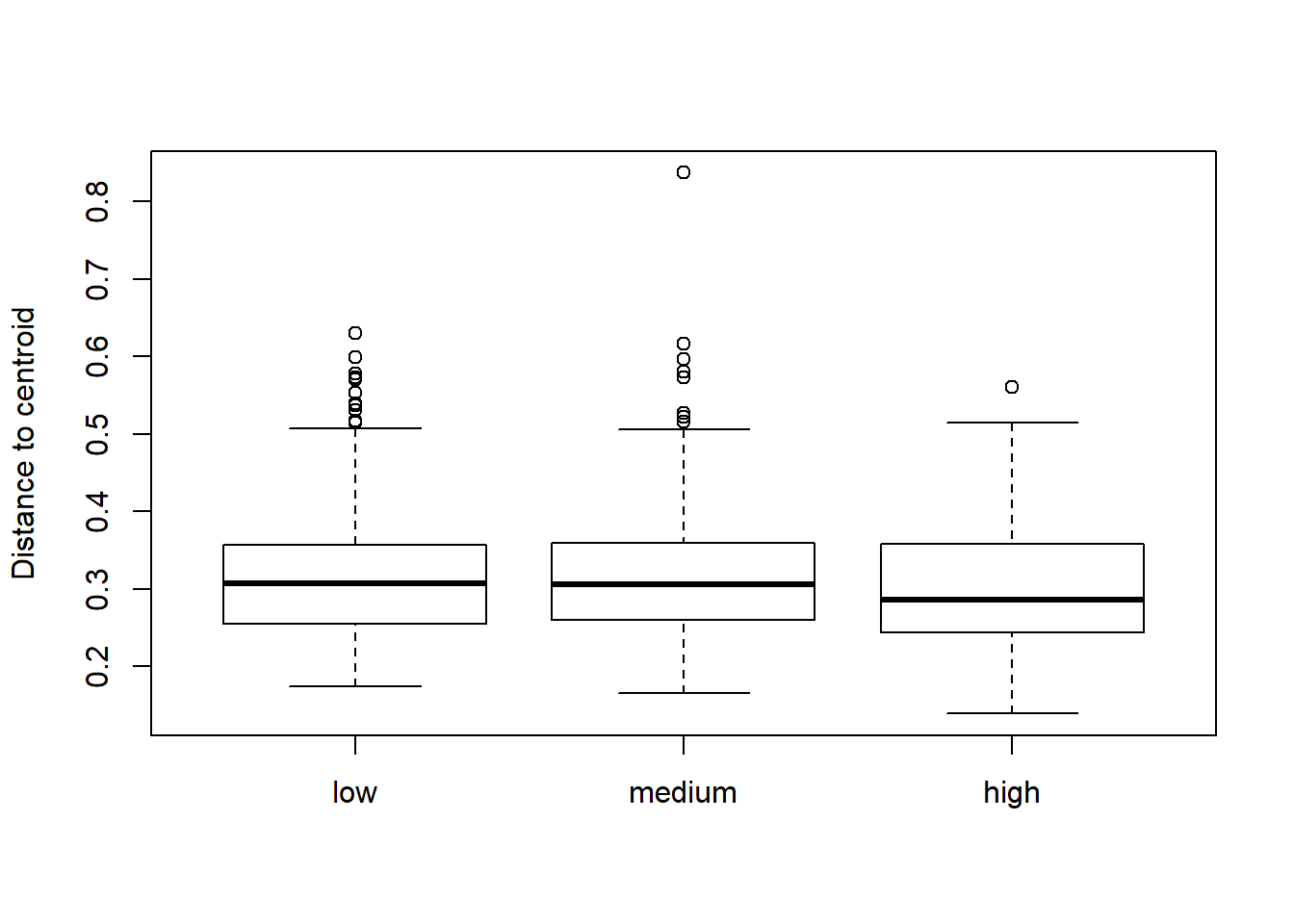 | 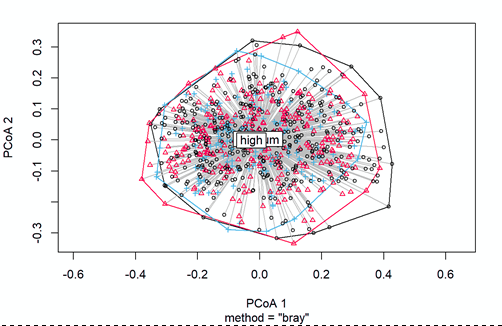 |
| Phenoxymethylpenicillin | 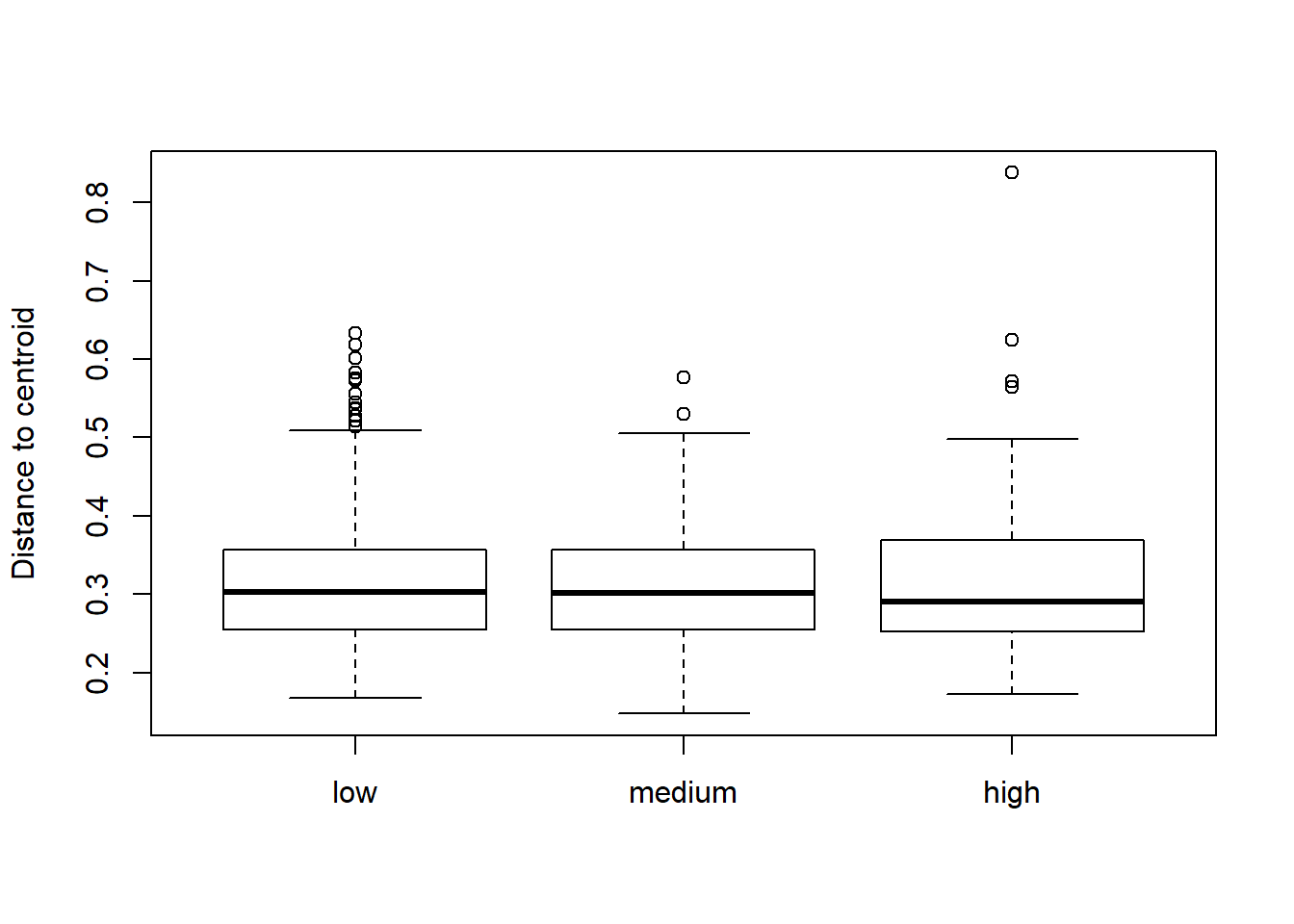 | 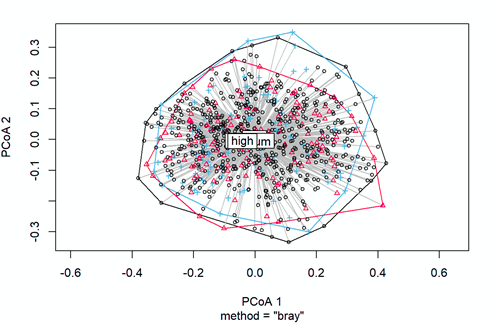 |

b)

| **AMs in boys** | **Distance to centroids** | **Beta dispersion (**Low, Medium, High**)** |
| --- | --- | --- |
| Amoxicillin-clavulanate | **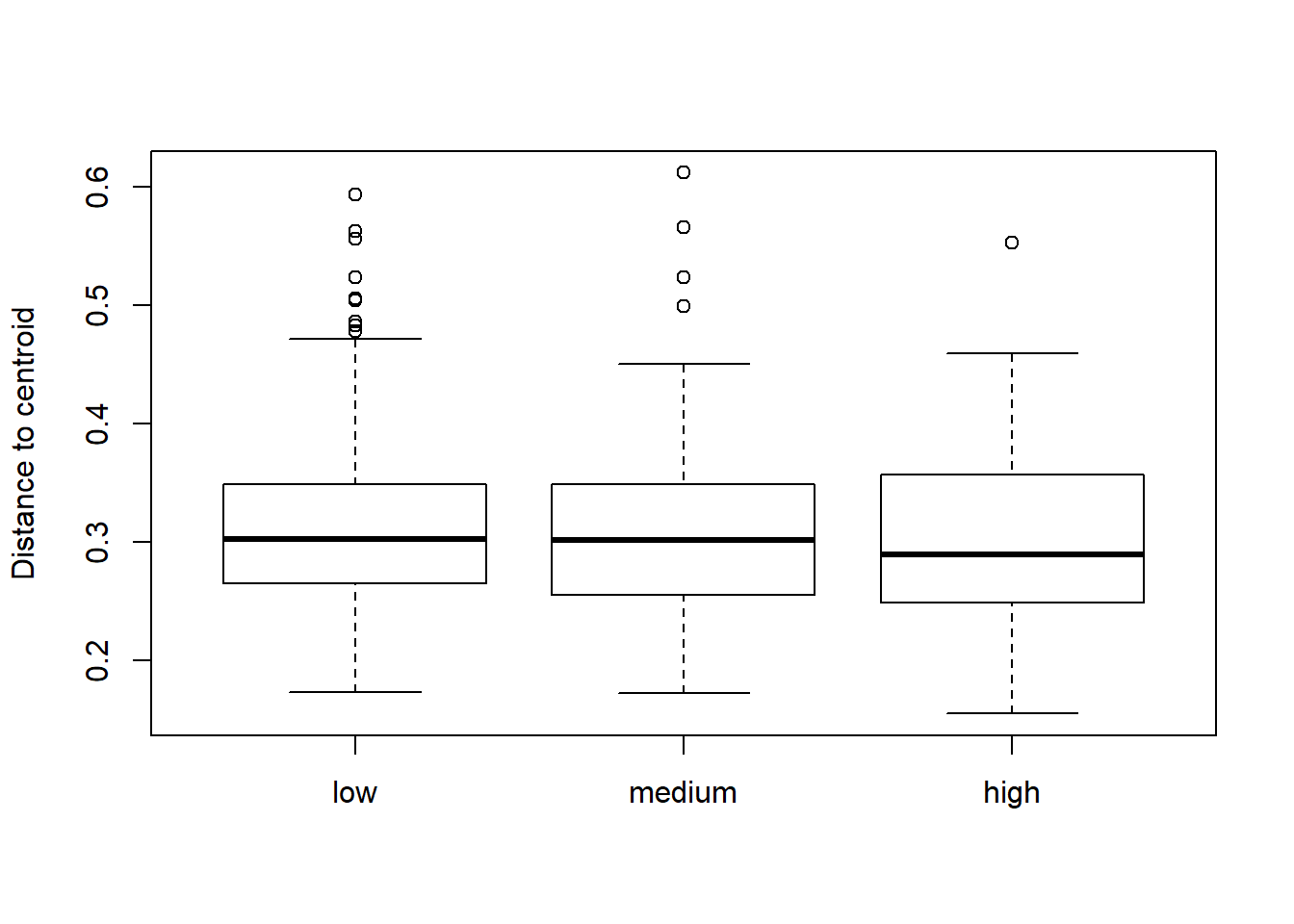** | 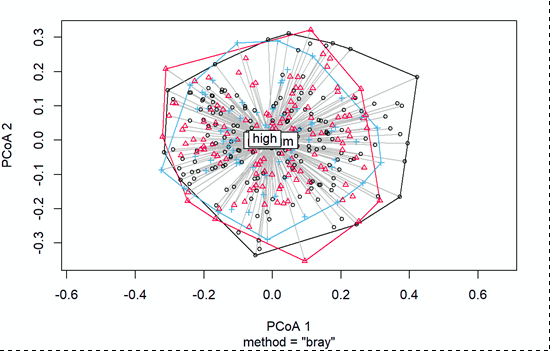l̥ |
| Phenoxymethylpenicillin | **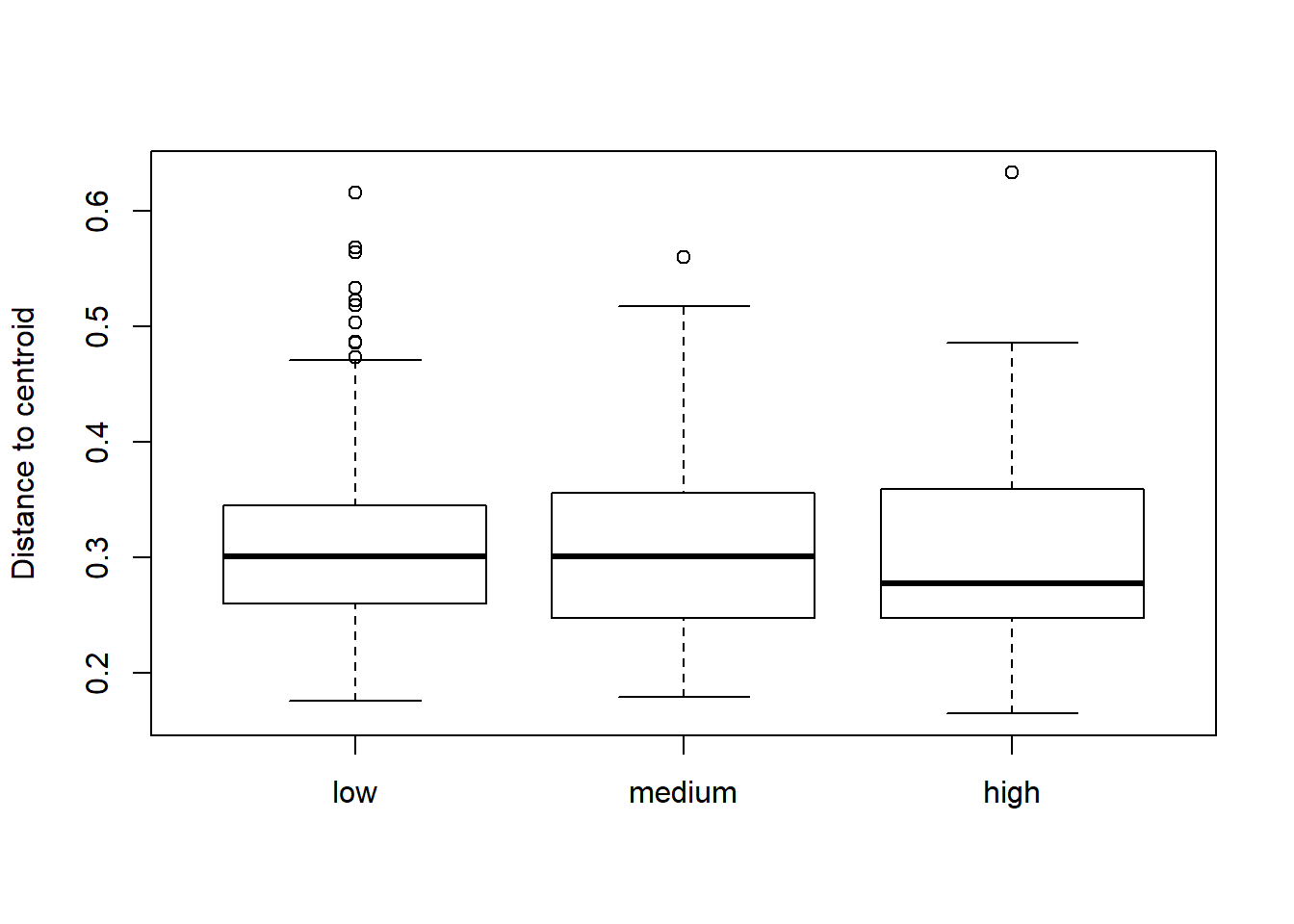** | 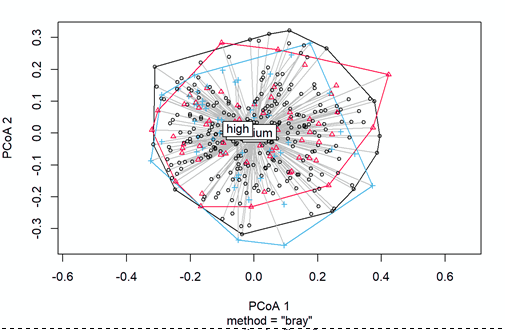 |

**c)**

| **AMs in girls** | **Distance to centroids** | **Beta dispersion (**Low, Medium, High**)** |
| --- | --- | --- |
| All AMs | **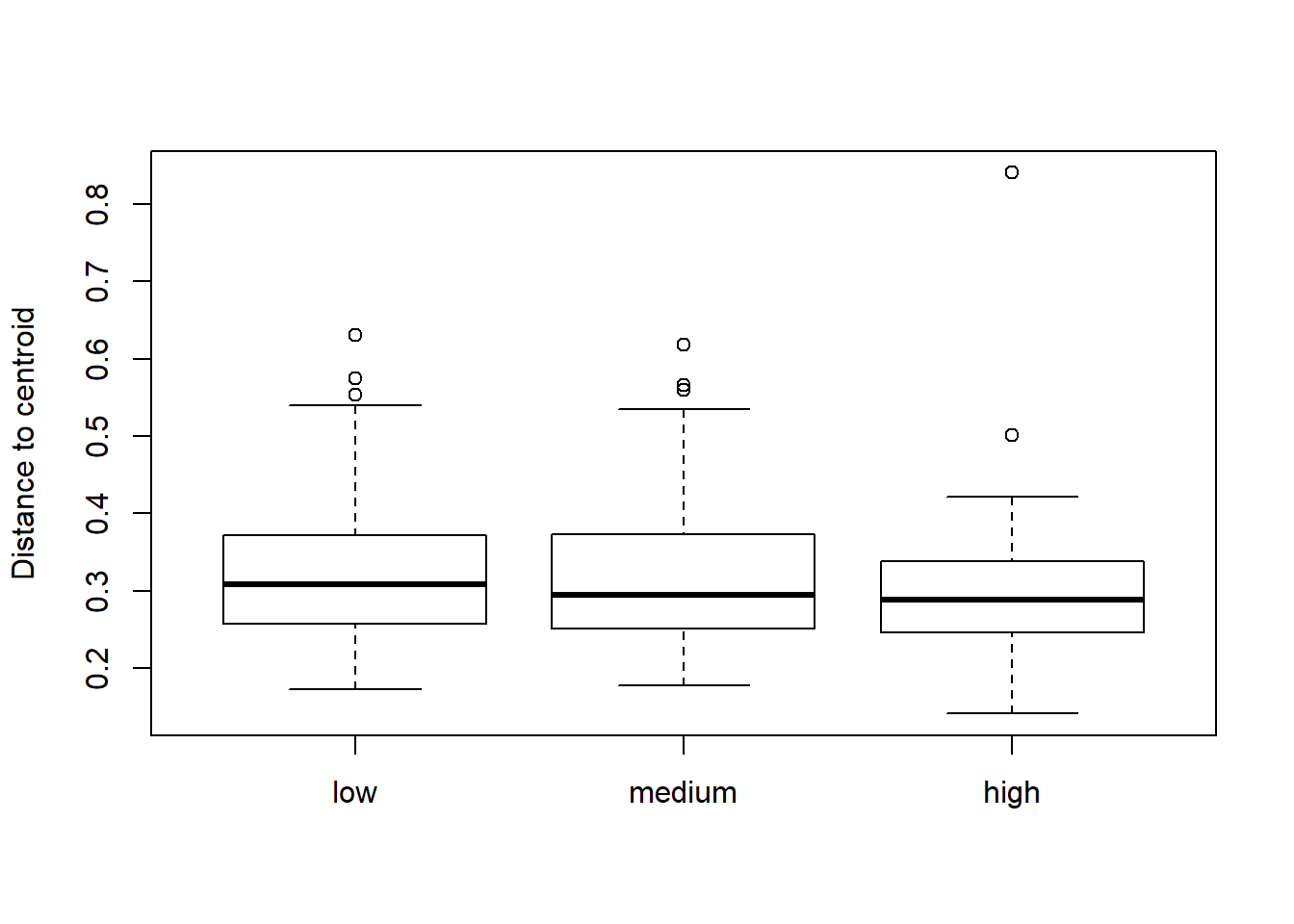** | 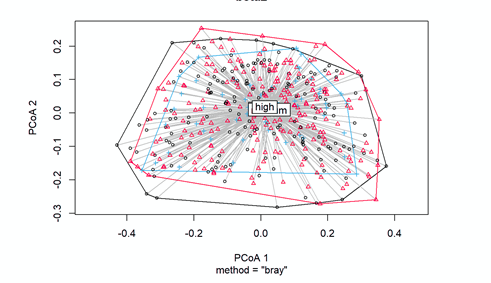 |
| Amoxicillin | **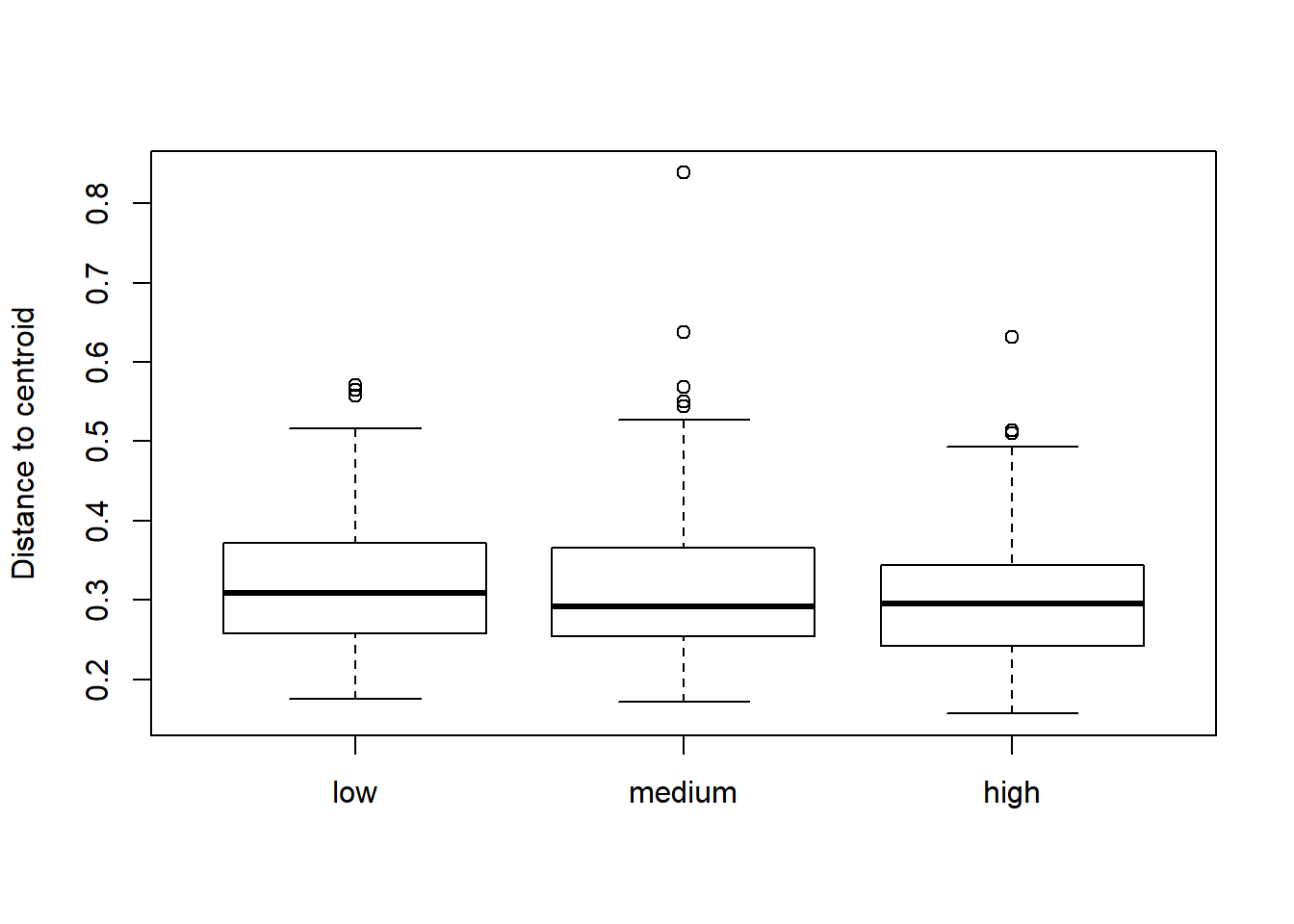** | 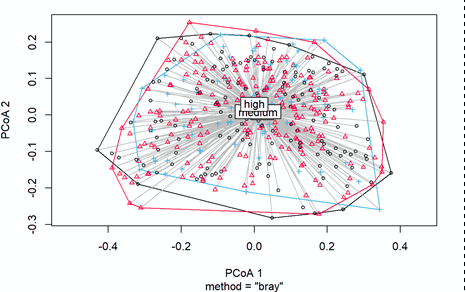 |
| Azithromycin | **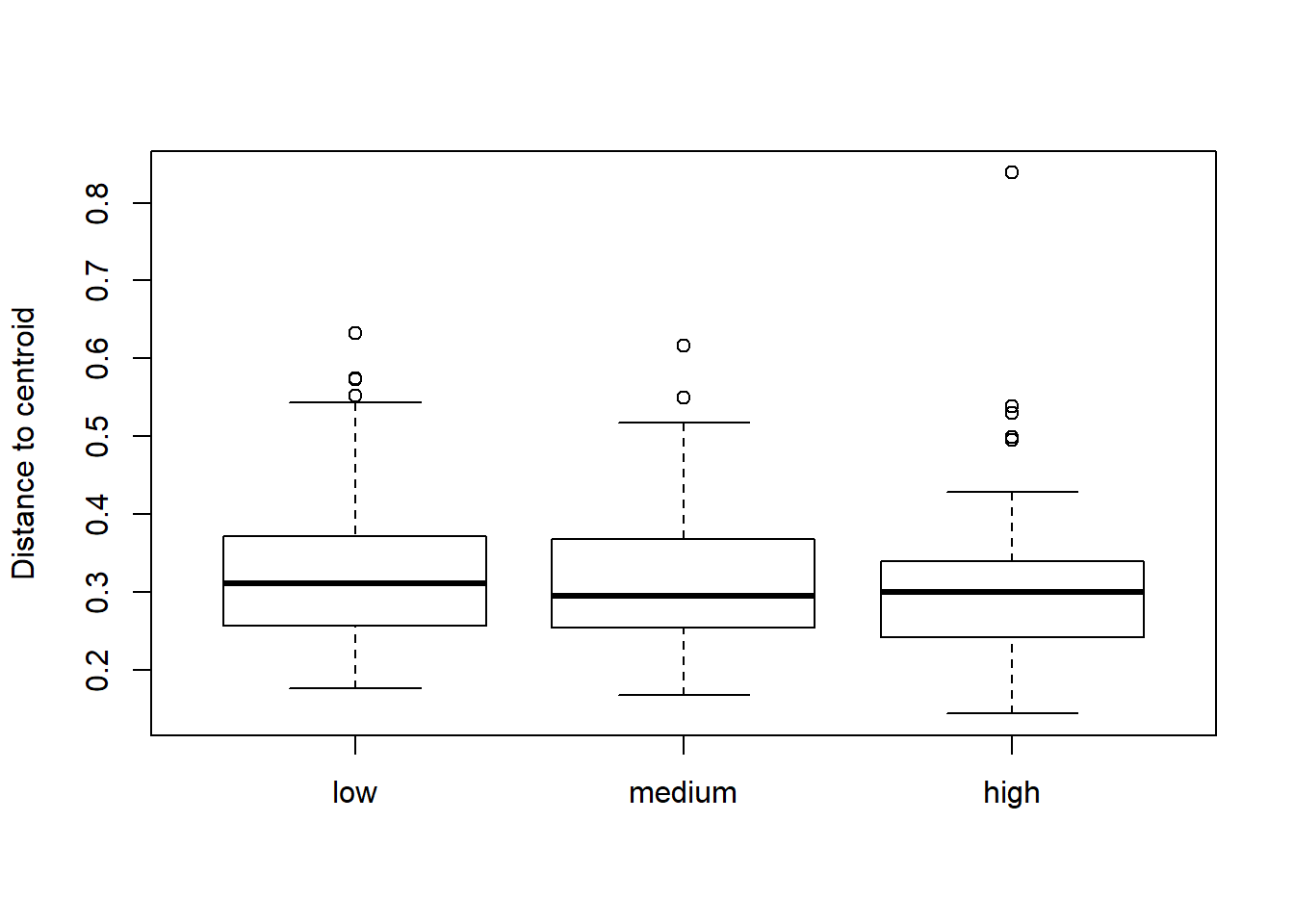** | 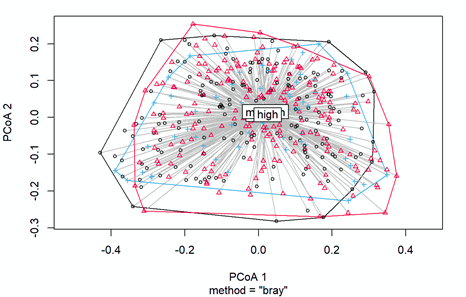 |
| Amoxicillin-clavulanate | **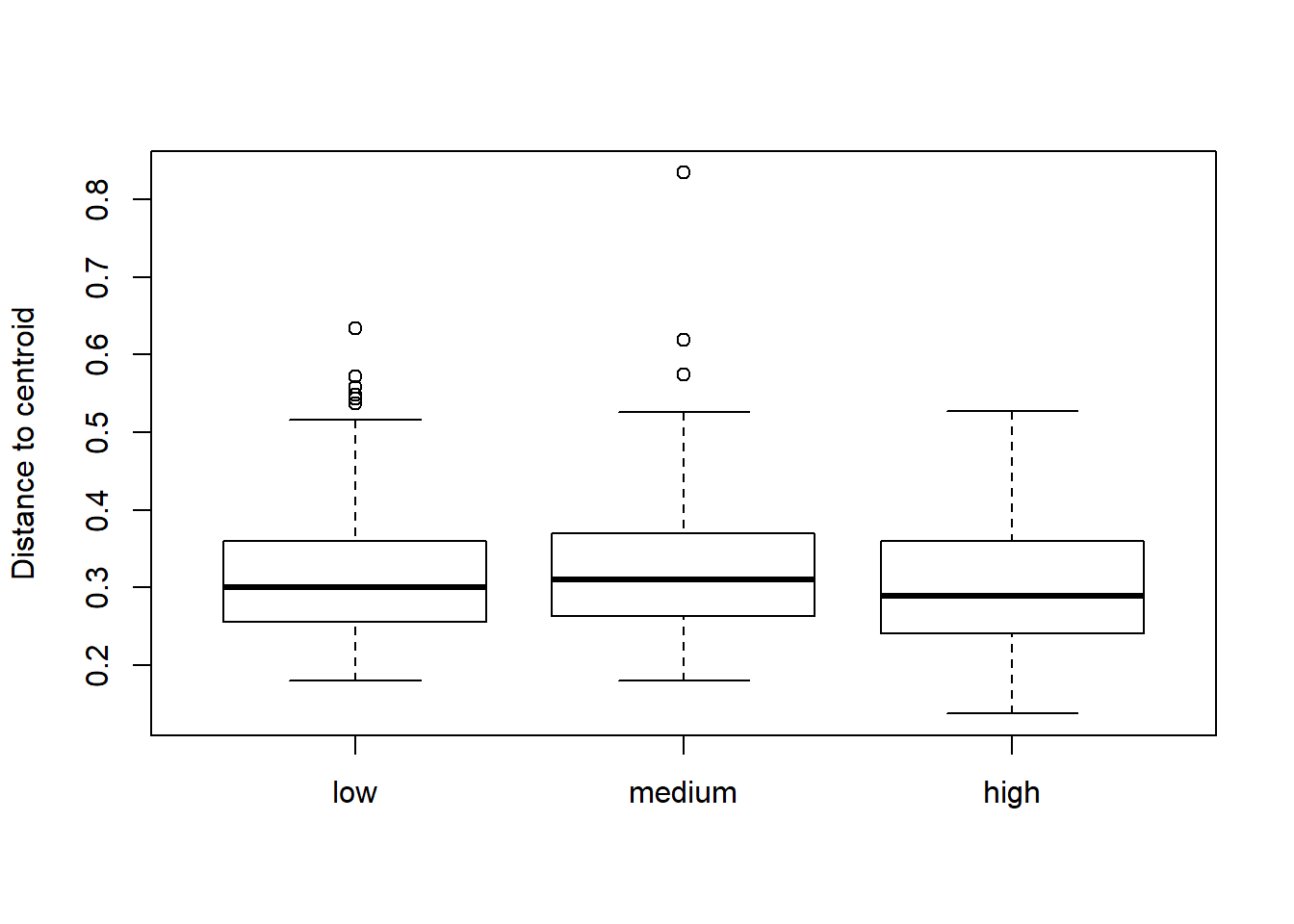** | 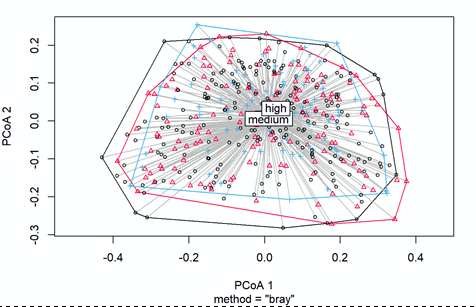 |
| Phenoxymethylpenicillin | **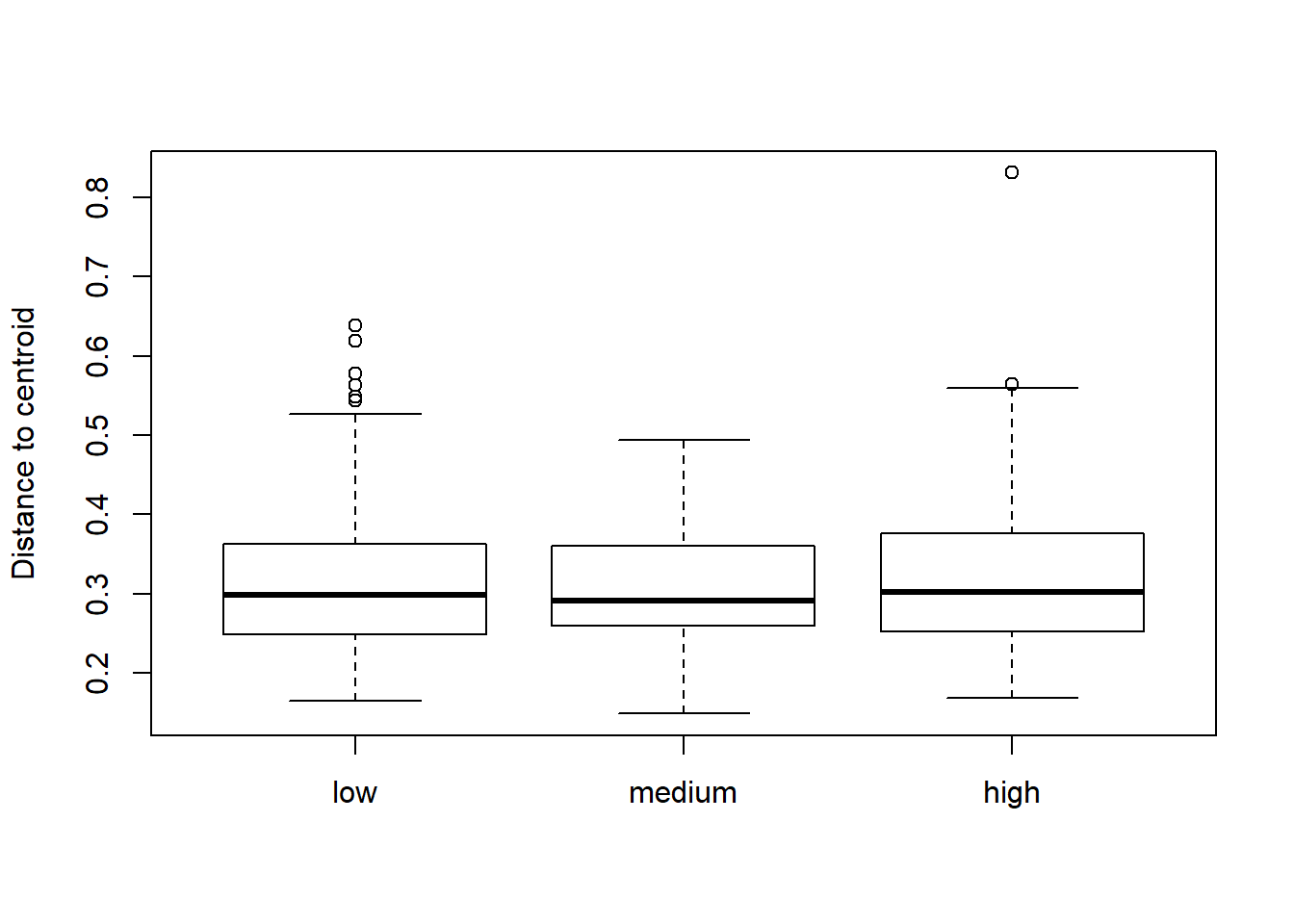** | 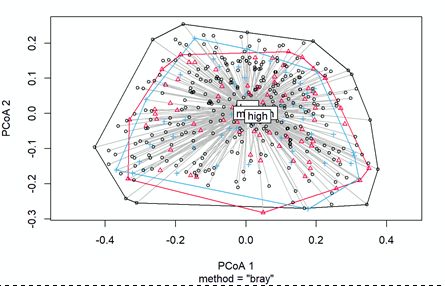 |
